# Foster Parents’ Parenting and the Social-Emotional Development and Adaptive Functioning of Children in Foster Care: A PRISMA-Guided Literature Review and Meta-Analysis

**DOI:** 10.1007/s10567-020-00336-y

**Published:** 2021-02-16

**Authors:** Sabrina Chodura, Arnold Lohaus, Tabea Symanzik, Nina Heinrichs, Kerstin Konrad

**Affiliations:** 1 Developmental Psychology and Developmental Psychopathology, University of Bielefeld, Bielefeld, Germany; 2 Clinical Psychology & Psychotherapy, University of Bremen, Bremen, Germany; 3 Child Neuropsychology Section, Dept. for Child and Adolescent Psychiatry, University Hospital RWTH Aachen, Aachen, Germany; 4 JARA-Brain Institute II, Molecular Neuroscience and Neuroimaging, RWTH Aachen & Research Centre Juelich, Juelich, Germany; 5 Department of Psychology, WU Developmental Psychology and Developmental Psychopathology, University of Bielefeld, P.O. Box 10 01 31, 33501 Bielefeld, Germany

**Keywords:** Foster children, Foster parents, Parenting, Meta-analysis, Literature review, Foster child development, Child development

## Abstract

Children in foster care (CFC) are at increased risk for negative developmental outcomes. Given the potential influence of foster parents’ parenting on the development of CFC, this literature review and meta-analysis provide an initial overview of how parenting factors in foster families relate to CFC’s developmental outcomes. We aimed to explore (1) whether foster parents’ parenting conceptualizations are related differently to various CFC developmental outcome variables and (2) how characteristics of foster parents and CFC moderate these associations. Following the recommendations of the PRISMA statement, we searched four databases in 2017 (with an update in May 2020). Forty-three primary studies were coded manually. The interrater agreement was 92.1%. Parenting variables were specified as parenting behavior, style, and goals and were distinguished further into functional and dysfunctional parenting. CFC development was divided into adaptive (including cognitive) development and maladaptive development. Meta-analyses could be performed for foster parenting behavior and developmental outcomes, as well as for functional parenting goals and maladaptive socioemotional outcomes in CFC. Associations between functional parenting behavior and adaptive child development were positive and negative for maladaptive child development, respectively. For dysfunctional, parenting effects were in the opposite direction. All effects were small to moderate. Similar results were found descriptively in the associations of parenting style and child developmental outcomes. We found similar effect sizes and directions of the associations between parenting behavior in foster families and the child’s developmental outcomes as those previously reported for biological families. These findings provide strong support for the significant role of parenting in foster families regarding children’s development in foster care.

**Supplementary Information:**

The online version of this article (10.1007/s10567-020-00336-y) contains supplementary material, which is available to authorized users.

## Introduction

### Children in Foster Care

Considering factors such as maltreatment experiences, former placement changes, and age at placement, the influence of foster parents’ parenting is fundamental for the development of children in foster care (CFC; Orme and Buehler 2001). Identifying important variables for effective foster parenting (i.e., parenting that facilitates adaptive child development, regulation, and adjustment, including cognitive, social-emotional functioning, and attachment security, and reduces maladjustment symptoms and developmental pathways) may help with the selection of appropriate foster parents for CFC (Washington et al. 2018). This can have positive effects on the development of CFC in particular and on the costs of youth welfare institutions in general.

Regarding the development of CFC, Leve et al. (2012) found that CFC with maltreatment experiences have a higher risk of developing mental disorders. They also often show developmental delays (Oswald et al. 2010). In addition, alterations in neuroendocrine stress-response functioning (Dozier et al. 2006; Fisher and Stoolmiller 2008) increase the risk of executive functioning deficits (Pears et al. 2008; Bruce et al. 2009), and alterations in social information processing and emotional regulation abilities (Price and Landsverk 1998; Kay et al. 2016) have been described in CFC across multiple studies. Maltreatment and neglect experiences of children may lead to adaptive behavior in an abusive family but may lead to problems in a foster family. For example, multiple studies have shown that children who have experienced maltreatment or abuse in the past are overly wary of angry faces (da Silva Ferreira et al. 2014). This might be adaptive when living with parents whose anger may be an important threat cue (Belsky et al. 2012); however, it comes at the cost of assuming hostile intent too readily under benign conditions and might thus lead to aggressive responses that would not have been evoked if the child’s attributions had been different (Dodge et al. 1995). Therefore, such behaviors may require tailored parenting responses among foster parents (Solomon et al. 2017).

This paper addresses the question of which kind of parenting dimensions are relevant to CFC’s developmental outcomes. It is based on a literature review and meta-analysis as important bases for systematically exploring the associations of parenting factors with the developmental outcomes in CFC.

### Definition of Parenting

Darling and Steinberg (1993) noted that it is essential to give a clear definition of the term “parenting” because of the use of different indicators for parenting in previous research. The authors introduced an integrative parenting model, which includes three different categories of parenting: parenting goals, parenting style, and parenting practices (or behavior). The present review follows these terms and definitions, as further explained below. Furthermore, we discriminate between *functional* and *dysfunctional parenting.* As a result, *functional parenting* can be seen within the dimensions of responsiveness and demandingness/control (e.g., Wolfe and McIsaac 2011), which includes provisions of warmth and limit setting matched to the children’s needs. This also includes the appropriate setting of boundaries on the one hand and encouraging children’s competencies on the other hand. By contrast, *dysfunctional parenting* is defined as either overly strict and rigid or unclear and inconsistent parental behavior, which therefore misses the appropriate orientation towards children’s needs. Furthermore, *dysfunctional parenting* includes the component of psychological control of children (i.e., parental attempts “to control their child’s behavior using psychological tactics aimed at undermining their emotional security or sense of self”; ibid., p. 804). *Parenting goals* include socialization goals for the child, such as the “acquisition of specific skills and behaviors” (e.g., appropriate manners or academic abilities) and of more global qualities (e.g., curiosity or critical thinking; Darling and Steinberg 1993, pp. 492–493). Therefore, parenting goals do not necessarily imply direct links to behavior, but instead refer to their fundamental attitudes towards parenting. *Parenting style* is defined as a “constellation of attitudes toward the child that are communicated to the child and create a specific emotional climate” (e.g., a combination of tone of voice and body language; ibid.). Baumrind (1991) identified four different kinds of parenting styles, as a result of combinations of parenting behavioral expressions. *Authoritative parents* show high responsiveness towards their child, and they practice appropriate control to encourage their child’s adaptive development. Parents with an *authoritarian* style show high expressions of appropriate control, as well as non-appropriate control (e.g., psychological control), to influence the child. They show low levels of responsiveness. High expressions of responsiveness without the use of any control characterize a *permissive* parenting style. Finally, *neglecting* parents show low levels of responsiveness and control. *Parenting behaviors* are “behaviors defined by specific content and socialization goals” (e.g., helping with homework and asking about hobbies; Darling and Steinberg 1993, pp. 492–493). Because of the immediate relation to specific situations, parenting behaviors are the easiest to observe.

### Parenting and Development of Children in Foster Care

Foster families are considered caring environments that enable the CFC to thrive despite past adverse experiences (Comas-Diaz et al. 2012). Thus, parenting in foster families might support resilience through role modeling processes and the experience of positive, trustworthy, and stable relationships. Schofield and Beek (2005) found qualitative data to support a successful foster family model in which foster parents’ parenting behavior promotes the trust of CFC in availability, reflective function, self-esteem, autonomy, and family membership. Moreover, Oswald et al. (2010) argued that even though there is much research on children in foster care regarding maltreatment, abuse, neglect, and mental health, very few studies have focused on the children’s long-term development after they transitioned into foster care.

### Associations Between Parenting and Children’s Development in Population-Based Studies

Regarding parenting and children’s development in the general population, meta-analyses showed small to moderate positive associations between functional parenting and adaptive child developmental variables and attachment security, respectively (Karreman et al. 2006; Pinquart 2015; De Wolff and van Ijzendoorn 1997). In contrast, dysfunctional parenting showed small to moderate negative associations with adaptive functioning (Karreman et al. 2006; Pinquart 2015). Additionally, externalizing and internalizing problem behaviors in children showed small to moderate negative associations with functional parenting (Rothbaum and Weisz 1994; Pinquart 2017) and small to moderate positive associations with dysfunctional parenting (Pinquart 2017; McLeod et al. 2007a, b).

Even though these results may give the first hint of how parenting and developmental outcomes relate to each other in foster families, it is challenging to answer this question based on these research results as they mainly come from studies that include biological families. On the one hand, foster parents’ parenting behavior might have a similarly strong, or even stronger association (compared to biological families) to the development of the CFC, given their adverse early life events (Heller et al. 1999). In addition, foster care placement is also often associated with a change in the *context of living* (e.g., by moving to an area with lower occurrences of community-level violence), which also influences children’s problem behaviors, and may thus increase the association between parenting and developmental outcomes in CFC (Lynch and Cicchetti 1998). Furthermore, it is considered particularly difficult to change experiences and learning processes during early childhood when they are adverse experiences. Additionally, children’s genetic predispositions and their parents’ childrearing regimes are known to be closely interwoven (Maccoby 2000). Thus, dissimilarities between the genetic make-up of foster parents and their nonbiological children might also contribute to smaller associations between parenting behavior and outcomes in these populations. McCrory and Viding (2015) proposed a latent vulnerability model that explains why children with maltreatment experiences more often show problem behavior and why interventions (such as living in a foster family) can be less effective for some children with maltreatment experiences than for others. The authors propose that persons with childhood maltreatment experiences adapt their neurocognitive threat processing, which may be vital in threatening environments. However, those persons may “overattribute threat in ways that increase the frequency of reactive aggression” (ibid., p. 500).

Furthermore, negative expectations of CFC regarding their foster parents’ behavior (because of adverse experiences in the biological family) may change the association between foster parents’ parenting and children’s development (Milan and Pinderhughes 2000), compared to the association between biological parents’ parenting and children’s behavior. This is in line with the results from Kemmis-Riggs et al. (2018). They found that more traditional parenting training for parents of biological children had little effect on foster families. Additionally, Gardenhire et al. (2019) noted that foster parents often miss information regarding CFC’s former experiences. This may also have a reducing effect on the association of foster parents’ parenting and the development of the CFC, because it is more difficult for foster parents to tailor their parenting in response to the needs of the CFC.

### Additional Factors Influencing the Relationship Between Parenting and Child Development

As indicated above, associations between developmental outcomes for children and parenting may be moderated by several proximal and distal factors. Such effects may have to be considered, especially in foster families. Several variables were identified regarding *child characteristics* that could influence foster care disruptions, which are supposed to be the result of maladaptive developmental pathways in CFC (Oosterman et al. 2007). As a result of this, CFC more often experienced disruptions when placed at an older age. Regarding the gender of the CFC, Leathers (2002) found a different prevalence of conduct problems for boys and girls in foster care, with boys showing fewer symptoms of conduct disorder. Additionally, placement instability is associated with more behavioral problems (Leathers 2002), developmental delays, and mental disorders in CFC (Oswald et al. 2010). However, because of the above noted assumed reciprocal longitudinal effects of parents’ and children’s behavior, the length of residence with the actual foster family may influence this association (ibid.).

Regarding *foster family characteristics,* the age of parents is one well-documented factor in the research of parenting (e.g., Van Holland De Graaf et al. 2018). Considering that there are often differences between the mean ages of foster and biological parents (Chodura et al. 2019), the age of foster parents is a potentially relevant variable for the association of parenting and development of CFC. Foster parents further can be differentiated into professional and nonprofessional foster caregivers. Professional foster caregivers often have special educational training (also called therapeutic foster care, e.g., Murray et al. 2010). In contrast, nonprofessional foster caregivers are not explicitly trained in these areas. However, they nevertheless often receive support from caseworkers. Oosterman et al. (2007) found that foster parents’ professionalism was associated with more adaptive developmental outcomes for CFC. Furthermore, Leslie et al. (2000) showed that the mental and physical needs of foster children are associated with the socioeconomic status (income, education, and employment status; APA, Task Force on Socioeconomic Status 2007) of the foster family and may moderate the associations between parenting and child development for CFC.

### Research Questions

Given the potentially challenging behaviors and special needs of CFC, it is crucial to investigate further the association between foster parents’ parenting and developmental outcomes of CFC. Although other reviews in the past have focused either on the efficacy of foster parents’ interventions as a whole (e.g., Goldman Fraser et al. 2013; Kerr and Cossar 2014; Kinsey and Schlösser 2013), different intervention components, or certain delivery formats (Kemmis-Riggs et al. 2018; Gubbels et al. 2019), or have descriptively summarized factors associated with outcomes for CFC (Jones et al. 2011; Goemans et al. 2015; Washington et al. 2018), none of them have attempted to provide a quantitative analysis of the associations between parenting behaviors, styles, and goals of foster parents and various outcome measures for CFC. Given the substantial individual and societal implications of foster care, it is of the highest importance to establish sound empirical evidence that improves the long-term developmental outcomes of this burdened population.

Therefore, in the present literature review and meta-analysis, we aim to answer the research questions of whether the parenting of foster parents is associated with the development of CFC, and more specifically, whether various components of foster parents’ parenting are differently related to specific developmental outcomes of CFC, including cognitive as well as social-emotional functioning. Furthermore, we aim to identify the characteristics of foster parents, the foster family, or the CFC that moderate these associations.

## Method

The meta-analysis strategy follows the Preferred Reporting Items for Systematic Reviews and Meta-Analyses (PRISMA) Statement (Moher et al. 2009). The review protocol is available in the supplemental electronic material.

### Search Strategy

**Fig. 1:**
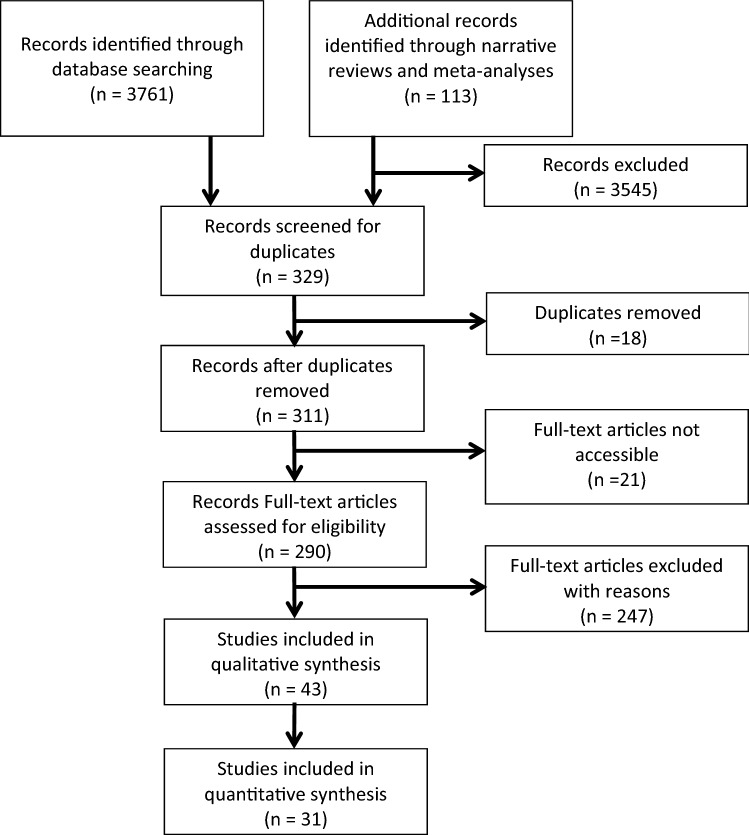
Flow diagram of study search

Figure 1 displays the research process. The initial literature search was done in August 2017 in the ERIC, PsycINFO, Psyndex, and PsycARTICLES databases. The following search terms were used in all variations over several search attempts: (foster children OR foster care) AND ([longitudinal OR repeated measures OR pretest posttest] OR [parent* OR rear* or care*]) AND relation*. No date limits were specified for the inclusion. The search was updated in May 2020. Overall, the search attempts led to 3761 potential publications. Then, the titles and abstracts of the publications were scanned. Full texts of conceivable studies were checked further for criteria regarding inclusion or exclusion. The lists of references of included articles were checked carefully for more potential primary studies, and 113 additional potential publications were found. Full texts were requested directly from the authors or purchased if not otherwise available on the various platforms.

For the search in 2017, authors of studies with missing data for the meta-analysis were contacted by email to obtain the missing data and ensure the inclusion of a maximum number of primary studies. Four additional studies were added by doing this. Twenty-one full texts could not be obtained online or by contacting the authors directly by mail; twenty of these were doctoral dissertations. In the 2020 update, all potentially interesting primary studies could be retrieved from the search platforms.

### Inclusion and Exclusion Criteria

Publications had to meet the following inclusion criteria: (a) *primary study about children in foster care*, (b) *outcome reflecting the children’s development or behavior*, (c) *at least one variable related to foster parents’ parenting*, (d) *study written in German or English*, (e) *sample group with at least ten participants,* and (f) *quantitative measures reported to compute an effect size.*

To increase the number of potential studies, and therefore, to increase the review’s representativeness, cross-sectional and longitudinal studies without treatment were both identified as appropriate. Waitlist or care-as-usual control groups in intervention studies were also identified as adequate study designs. It was assumed that the results of control groups (without the results of the intervention groups) would show longitudinal pathways, as seen in other studies without interventions. Studies investigating CFC samples with medical conditions (e.g., substantiated prenatal drug exposure) were excluded due to potential biases.

The reasons for excluding a primary study were mostly related to “parenting not examined” (*N* = 104), “a qualitative design of the primary study” (*N* = 38), or “not enough quantitative information” (*N* = 37).

### Coding Decisions and Computation of Effect Sizes

The coding manual for the meta-analytical computation consisted of two variable areas: one in which the moderators that had been shown in earlier research to potentially influence the parenting-development association were included. Coded variables for this area were *Children in foster care:* the examined area of development of the CFC, mean age of the CFC, the gender distribution of the CFC, length of residence in the current foster family, and the number of placements of the CFC; *Foster parents:* area of parenting (parenting goals, parenting style, parenting behavior; functional vs. dysfunctional), and the mean age of the foster parents; *Foster Families:* professionalism, kinship relationships between the foster parents and the CFC, the number of children living in the family, the family income, the highest education level of the foster parents, and their employment status.

The second area contained general study descriptions and potential study artifacts that may also affect the reported associations. These consisted of *General characteristics*: the publication year, authors, scientific area, country of study, and publication type; *Children in foster care:* the information source; *Foster parents:* the information source; *Measurements*: the study design, the statistical measurement of the association, the sample size, the time between measurements (for longitudinal studies), and an examination of additional variables; *Study quality.* The study quality estimation followed the Strengthening the Reporting of Observational Studies in Epidemiology (STROBE) Statement (von Elm et al. 2014). For every item on the primary study checklist, it was rated (*yes* = 1, *no* = 0) in terms of whether the primary authors followed the recommendations. Studies could achieve a maximum of 22 points according to the STROBE statement’s list of recommendations (ibid.). The complete coding manual can be found in the supplemental materials related to this article.

Two independent raters did the coding following a coding manual generated by the first author. One study (Ackerman and Dozier 2005) was coded by both raters and then discussed to ensure the coding sheet’s unambiguity. Differences and misunderstandings were deliberated before coding the rest of the studies. This study, therefore, was not included in the computation of interrater agreement. Interrater agreement across all variables in the computation was 92.06% and therefore excellent. It was lower but still satisfactory for *coding of additional variables* (72.29%), *area of parenting* (75.80%), and *area of child development* (86.94%). The agreement was highest for *type of measurement* and *professionalism* (99.36% each), as well as for *publication year* and *study design* (98.41% each).

#### Foster Parents’ Parenting

*Functional* and *dysfunctional parenting* was defined following the theoretical assumptions of the primary study authors. Wolfe and McIsaac (2011) discussed the possibility of viewing functional and dysfunctional parenting as parts of one dimension of parenting. However, we decided to examine both parenting dimensions separately for three reasons. First, parenting was often examined in primary studies from one of the above-discussed dimensions (responsiveness, demandingness/control, psychological control). Therefore, a combination in one variable may be biased by an overrepresentation of one dimension. Second, functional and dysfunctional parenting may both occur in one parent. For example, a parent may show interest in a child’s hobby (*functional behavior*) and also use corporal punishment for a child’s misconduct (*dysfunctional behavior*). Finally, functional and dysfunctional parenting behaviors are mostly defined by research from community samples. At this point, we do not know if those parenting dimensions account for foster families as well.

Additionally, foster parents’ emotional investment (Ackerman and Dozier 2005), as well as foster parents’ sensitivity (e.g., Ponciano 2010), as parts of parental attachment behavior, are often distinctly conceptualized from parenting behavior. However, those concepts are measured by examining parenting behavior, defined by specific content and socialization goals, as Darling and Steinberg (1993) described for parenting behavior. Therefore, the parents’ attachment behavior was interpreted as *functional parenting behavior* that promotes the development of secure attachment and adaptive functioning in children (e.g., Ackerman and Dozier 2005). By this, we followed the definitions of authors of the primary studies as well. Although some constructs (e.g., parental criticism or overprotection) might also be differently classified in another category (e.g., parenting style vs. parenting goals), we decided not to change the primary classification derived from the systematic search criteria based on Darling and Steinberg’s (1993) definitions of parenting. This approach allows the calculation of effect sizes across clearly defined sets of studies, improving our findings’ reproducibility in future meta-analyses.

Therefore, for the coding of parenting, the following categories were used: functional and dysfunctional parenting goals, functional and dysfunctional parenting styles, and functional and dysfunctional parenting behaviors. Each parenting variable was allocated to only one of the categories.

#### Developmental Outcomes of Children in Foster Care

We used broad definitions for developmental outcomes in line with the meta-analysis of Goemans et al. (2015), which focused on adaptive functioning (defined as “meeting age and culturally appropriate standards of personal independence and social functioning,” p. 122), internalizing (“problems that primarily affect a person him or herself,” p. 122), externalizing (“problems that primarily affect a person’s social environment,” p. 122), and total problem behavior (the emergence of both internal and external problems) regarding children’s development. Furthermore, we also included cognitive developmental outcomes for CFC. However, cognitive outcomes were scarcely examined in relation to parenting dimensions, which is why we subsumed these studies under the category of adaptive functioning. We also decided to consider specific outcomes for CFC as well, such as placement disruptions. Additionally, although not specified in the search terms, *attachment security* was often examined. This was why it became an additional category for the development of CFC. Consequently, the following categories for CFC development were coded: adaptive functioning, internalizing problems, externalizing problems, attachment security, placement stability, and total problem behavior.

### Statistical Analyses

The effect sizes were computed with the statistics program *R* (R Core Team 2017) using the packages *metafor* (Viechtbauer 2010) and *robumeta* (Fisher et al. 2017). The moderator analyses were done as meta-regressions with *robumeta.* A random-effects model was conducted (Hedges 1981). Although there was no restriction for publication type, it was checked for publication bias by the trim-and-fill method (Duval and Tweedie 2000). This method assumes that funnel plots will show left and right symmetrical variance from the overall effect size of a meta-analysis if there is no publication bias. It, therefore, shows how many studies need to be reported to obtain the assumed symmetry. In some studies, sample sizes of CFC and foster parents were different, so the sample size of CFC was included in the analysis.

In some studies, the same construct was presented with different outcomes or independent variables. Therefore, effect sizes were integrated through *robumeta* to clusters according to the recommendation of Hedges et al. (2010). Following this step, an intercorrelation *ρ* had to be estimated to define clusters. A conservative correlation of *ρ*  = 0.99 between studies of the same cluster was assumed in the study at hand. Clusters had been defined when effect sizes were provided in the same study or when characteristics (e.g., authors, sample sizes, and descriptions) in studies showed that the same sample was used for different papers. Therefore, the results can be reported on the *variable level* or *study level.* The *variable level* results were all integrated according to effect sizes, which could be more than one per study, corrected by clustering. The results on the *study level* (e.g., descriptions of the studies) were based on the number of studies. Effect sizes were interpreted according to Cohen (1988), with *r* = 0.1 as small, *r* = 0.3 as moderate, and *r* = 0.5 as high effect.

## Results

Overall, 43 studies with 314 effect sizes were included. A quantitative meta-analysis was possible for the association between parenting behavior and children’s development (*k* = 31 studies). By contrast, for parenting style (*k* = 4 studies), there were not enough studies for a meta-analysis. For parenting goals (*k* = 12 studies), meta-analyses could be conducted only for functional parenting goals and internalizing and externalizing behaviors. We report a qualitative summary for the parenting dimensions when computing a meta-analysis was not possible.

Overall, the publication years ranged from 1994 to 2019, with sample sizes of the CFC from *n* = 20 to *n* = 5516 (Md = 64). Most primary studies (67.44%) were conducted in the United States. Nine of forty-three studies were longitudinal (20.93%). Psychology was the research area that was most often declared (55.81%). An overview of the characteristics of the studies and the variables within the study levels can be seen in Table 1. The main characteristics of the variables are summarized in Tables E1 and E2 in the supplemental materials.

**Table 1:** Overview of studies included in the review

Part 1									
Study	Science area	Country	Publication type	Study design	T. b. M.^2^ (days)	S. Q.^3^	Measure type	E. A. V.^4^	S. S. CFC^5^
Ackerman and Dozier (2005)	Psychology	USA	Journal Article	Cross-sectional	–	14	Association	Yes	39
Bovenschen et al. (2016)^1^	Psychology	Other	Journal Article	Cross-sectional	–	18	Association	Yes	49
Chesmore et al. (2017)^1^	Other	USA	Journal Article	Cross-sectional	–	19	Association	Yes	493
Cooley et al. (2015)	Other	USA	Journal Article	Cross-sectional	–	16	b-weight	Yes	155
De Robertis and Litrownik (2004)	Other	USA	Journal Article	Cross-sectional	–	19.5	b-weight	Yes	70
De Schipper et al. (2012)^1^	Other	Other	Journal Article	Cross-sectional	–	20	Association	Yes	59
DeLisle (2011)^1^	Other	USA	Dissertation	Longitudinal	1440	17	Association	No	188
Denuwelaere and Bracke (2007)^1^	Other	Other	Journal Article	Cross-sectional	–	19	b-weight	Yes	96
Dubois-Comtois et al. (2015)^1^	Psychology	Other	Journal Article	Cross-sectional	–	19	b-weight	Yes	83
Estep (2008)	Psychology	USA	Dissertation	Cross-sectional	–	17	b-weight	Yes	103
Fuentes et al. (2015)^1^	Psychology	Other	Journal Article	Cross-sectional	–	16	Association	Yes	104
Gabler et al. (2014)^1^	Psychology	Other	Journal Article	Longitudinal	180	18.5	Association	Yes	48
Harden et al. (2015)^1^	Other	USA	Journal Article	Cross-sectional	–	18	b-weight	No	47
Harden et al. (2014)^1^	Other	USA	Journal Article	Cross-sectional	–	16	Association	Yes	63
Harden et al. (2017)^1^	Other	USA	Journal Article	Cross-sectional	–	18.5	Association	Yes	50
Harpin et al. (2013)^1^	Other	USA	Journal Article	Cross-sectional	–	17	b-weight	Yes	5516
Heywood (2009)^1^	Other	USA	Dissertation	Longitudinal	1460	16	Association	Yes	24
Jacobsen et al. (2018)	Psychology	Other	Journal Article	Cross-sectional	–	18.5	b-weight	No	60
Jones (2004)^1^	Psychology	USA	Dissertation	Cross-sectional	–	17	Association	Yes	108
Kelly (2015)^1^	Psychology	Other	Dissertation	Cross-sectional	–	16.5	Association	Yes	20
Leon et al. (2008)^1^	Psychology	USA	Journal Article	Longitudinal	548	16	b-weight	Yes	142
Linares et al. (2006)^1^	Other	USA	Journal Article	Cross-sectional	–	16	b-weight	Yes	64
Lindhiem and Dozier (2007)	Psychology	USA	Journal Article	Longitudinal	324	19	Association	Yes	82
Migliorini et al. (2016)^1^	Other	Other	Journal Article	Cross-sectional	–	16	Association	No	48
Miller et al. (2019)	Psychology	Other	Journal Article	Longitudinal	600	17.5	Cohen's *d*	Yes	75
Olson et al. (2019)^1^	Psychology	USA	Journal Article	Longitudinal	180	19	Association	Yes	91
Oosterman and Schuengel (2008)^1^	Other	Other	Journal Article	Cross-sectional	–	16	Association	No	47–61
Perkins (2008)^1^	Psychology	USA	Journal Article	Cross-sectional	–	15	Association	No	143
Perkins and Flynn (2008) ^1^	Psychology	USA	Journal Article	Cross-sectional	–	18	Association	No	439
Ponciano (2010)^1^	Other	USA	Journal Article	Cross-sectional	–	15	Association	Yes	76
Ponciano (2012)^1^	Other	USA	Journal Article	Cross-sectional	–	13	Association	Yes	76
Richardson and Gleeson (2012)	Other	USA	Journal Article	Cross-sectional	–	16.5	b-weight	Yes	120
Rogers (2016)	Psychology	USA	Dissertation	Cross-sectional	–	18	Association	Yes	33/34
Salas et al. (2015)^1^	Psychology	Other	Journal Article	Cross-sectional	–	21.5	Association	Yes	104
Sandow (1998)^1^	Psychology	USA	Dissertation	Cross-sectional	–	17	Association	No	42
Schofield (2010)	Psychology	USA	Dissertation	Cross-sectional	–	12	Association	Yes	44
Smith (1994)^1^	Other	USA	Journal Article	Cross-sectional	–	18	Association	No	38
Somers (2010)	Psychology	USA	Dissertation	Cross-sectional	–	21	Association	Yes	100
Stovall (2001)	Psychology	USA	Dissertation	Longitudinal	60	16	t-value	Yes	38
Tucker (2011)^1^	Psychology	USA	Dissertation	Cross-sectional	–	16	Association	Yes	86
Vanderfaeillie et al. (2012)^1^	Psychology	Other	Journal Article	Longitudinal	730	19	Association	Yes	49
Vasileva and Petermann (2017)^1^	Psychology	Other	Journal Article	Cross-sectional	–	19	Association	Yes	286
Vuchinich et al. (2002)^1^	Other	USA	Journal Article	Cross-sectional	–	19	Association	Yes	23

Part 2							
Study	Area(s) of child development	Information source(s) for child development	Area(s) of parenting	Dimension(S) of parenting	Information source for parenting	M. A. CFC^7^	G. CFC^8^
Ackerman and Dozier (2005)	Total Problem Behaviors	External Report	Functional parenting goals	Responsiveness	Self Report	2.4 (0.92)	54
Bovenschen et al. (2016)^6^	Attachment Security	Behavioral observation	Functional parenting behavior	Responsiveness	Behavioral observation	5.51 (1.55)	49
Chesmore et al. (2017)^6^	Internalizing Problems	External Report, Self Report	Functional parenting behavior	Responsiveness	External Report	10,4 (0,9)	51,1
Cooley et al. (2015)	Externalizing Problems	External Report	Dysfunctional parenting goals	Psychological Control	Self Report	n/a	n/a
De Robertis and Litrownik (2004)	Externalizing Problem Behaviors	External Report, Self Report	Dysfunctional parenting behavior	Psychological Control	Self Report	8 (n/a)	52.9
De Schipper et al. (2012)^6^	Attachment Security	External Report	Functional parenting behavior	Responsiveness	Behavioral observation	4.75 (1.37)	37
DeLisle (2011)^6^	Internalizing & Externalizing Problem Behaviors	External Report, Self Report	Functional parenting behavior	Responsiveness & Demandingness	External Report	13 (1.36)	40
Denuwelaere and Bracke (2007)^6^	Internalizing & Externalizing Problem Behaviors	Self Report	Functional parenting behavior	Responsiveness	External Report	14.5 (3.1)	48.4
Dubois-Comtois et al. (2015)^6^	Internalizing & Externalizing Problem Behaviors	External Report	Functional parenting behavior & goals	Responsiveness	Self Report	5.13 (1.76)	62.7
Estep (2008)	Placement Stability	External Report	Functional & dysfunctional parenting style	Demandingness & Psychological Control	Self Report	n/a	n/a
Fuentes et al. (2015)^6^	Internalizing, Externalizing, and Total Problem Behaviors	External Report	Functional & dysfunctional parenting behavior & style	Responsiveness, Demandingness, & Psychological Control	Self Report	11 (3.2)	53.8
Gabler et al. (2014)^6^	Attachment Security	Behavioral observation	Functional & dysfunctional parenting behavior	Responsiveness & Psychological Control	Behavioral observation	2.6 (1.44)	50
Harden et al. (2015)^6^	Adaptive Functioning	Behavioral observation	Functional parenting behavior	Responsiveness	Behavioral observation	5.08 (0.83)	n/a
Harden et al. (2014)^6^	Adaptive Functioning	Self Report	Functional parenting behavior	Responsiveness	Behavioral observation	5.3 (0.83)	n/a
Harden et al. (2017)^6^	Adaptive Functioning	Behavioral observation	Functional & dysfunctional parenting behavior	Responsiveness, Demandingness, & Psychological Control	Behavioral observation	5.3 (0.8)	48
Harpin et al. (2013)^6^	Internalizing Problems	Self Report	Functional parenting behavior	Responsiveness	External Report	14.55 (n/a)	55.4
Heywood (2009)^6^	Adaptive Functioning and Attachment Security	External Report	Dysfunctional parenting behavior	Psychological Control	Self Report	3.94 (0.75)	57.1
Jacobsen et al. (2018)	Internalizing & Externalizing Problems, Adaptive Functioning	External Report	Functional parenting goals	Responsiveness	Self Report	1.94 (0.05)	37.5
Jones (2004)^6^	Internalizing, Externalizing, and Total Problem Behaviors	External Report	Functional parenting behavior	Responsiveness	Self Report	9.4 (2.3)	58.4
Kelly (2015)^6^	Total Problem Behaviors	External Report	Functional parenting behavior & goals	Responsiveness & Demandingness	Behavioral observation, Self Report	7.88 (3.36)	42
Leon et al. (2008)^6^	Internalizing Problems	Self Report	Functional parenting behavior	Responsiveness & Demandingness	External Report	13.2 (1.9)	73
Linares et al. (2006)^6^	Externalizing Problems	External Report	Functional & dysfunctional parenting behavior	Demandingness & Psychological Control	Self Report	6.2 (2.3)	47
Lindhiem and Dozier (2007)	Total Problem Behaviors	External Report	Functional parenting goals	Responsiveness	Self Report	1.85 (0.9)	53,9
Migliorini et al. (2016)^6^	Internalizing, Externalizing, and Total Problem Behaviors	External Report	Functional parenting behavior & goals	Demandingness	Self Report	11 (4.8)	n/a
Miller et al. (2019)	Placement Stability	External Report	Functional parenting goals	Responsiveness	Self Report	9.96 (3.81)	53,3
Olson et al. (2019)^6^	Total Problem Behaviors	External Report	Dysfunctional parenting behavior	Psychological Control	Self Report	2.26 (0.44)	56
Oosterman and Schuengel (2008)^6^	Internalizing & Externalizing Problems, Attachment Security	Behavioral observation, External Report	Functional parenting behavior	Responsiveness	Behavioral observation	4.7 (1.38)	36.1
Perkins (2008)^6^	Internalizing & Externalizing Problems, Attachment Security	Self Report	Functional & dysfunctional parenting behavior	Responsiveness, Demandingness, & Psychological Control	Self Report	13.65 (1.65)	55
Perkins and Flynn (2008) ^6^	Internalizing & Externalizing Problems, Attachment Security	Self Report	Functional & dysfunctional parenting behavior	Responsiveness, Demandingness, & Psychological Control	Self Report	13.59 (2.16)	50
Ponciano (2010)^6^	Adaptive Functioning	Behavioral observation	Functional parenting behavior	Responsiveness	Behavioral observation	1.87 (0.7)	50
Ponciano (2012)^6^	Adaptive Functioning	External Report	Functional parenting behavior	Responsiveness	Behavioral observation	1.87 (0.7)	50
Richardson and Gleeson (2012)	Externalizing Problems	External Report	Functional parenting behavior	Responsiveness & Demandingness	Self Report	13.16 (n/a)	47
Rogers (2016)	Placement Stability	External Report	Functional & dysfunctional parenting style	Responsiveness, Demandingness, & Psychological Control	Self Report	Non-kin = 13.21 (4.08) Kin = 11.44 (3.39)	Non-kin = 57.6 Kin = 50
Salas et al. (2015)^6^	Total Behavior Problems & Impulsivity/Inattention	External Report	Functional & dysfunctional parenting behavior, dysfunctional parenting goals	Responsiveness, Demandingness, & Psychological Control	Self Report	11 (3.2)	53.8
Sandow (1998)^6^	Internalizing & Externalizing Problems, Adaptive Functioning	External Report	Functional & dysfunctional parenting behavior	Responsiveness	External Report	n/a	52.4
Schofield (2010)	Internalizing & Externalizing Problems, Adaptive Functioning	External Report	Functional parenting goals	Responsiveness	Self Report	5.25 (2.08)	59
Smith (1994)^6^	Internalizing & Externalizing Problems, Adaptive Functioning	External Report	Functional parenting behavior & styles	Responsiveness & Demandingness	Behavioral observation	4.44 (n/a)	53
Somers (2010)	Placement Stability	External Report	Functional & dysfunctional parenting goals	Responsiveness & Psychological Control	Self Report	4.02 (n/a)	n/a
Stovall (2001)	Attachment Security	External Report	Functional parenting goals	Responsiveness	Self Report	1.06 (0.42)	60
Tucker (2011)^6^	Adaptive Functioning, Internalizing & Externalizing Problems	External Report	Functional & dysfunctional parenting behavior	Demandingness & Psychological Control	Self Report	6.71 (1.2)	48.8
Vanderfaeillie et al. (2012)^6^	Internalizing, Externalizing, & Total Problem Behavior	External Report	Functional & dysfunctional parenting behavior	Responsiveness, Demandingness, & Psychological Control	Self Report	9.3 (1.7)	36.7
Vasileva and Petermann (2017)^6^	Internalizing & Externalizing Problems	External Report	Functional & dysfunctional parenting behavior	Psychological Control	Self Report	4.83 (1.15)	50.7
Vuchinich et al. (2002)^6^	Internalizing & Externalizing Problem Behaviors	External Report	Functional & dysfunctional parenting behavior	Responsiveness & Psychological Control	Behavioral observation	13.57 (2.35)	52.2

Part 3									
Study	L. R.^10^	M. A. F. P.^11^	N. S.^12^	N. P.^13^	Kin Care	Prof. F.P.^14^	F. F. I.^15^	Highest education^16^	Employment state^16^
Ackerman and Dozier (2005)	19	n/a	n/a	n/a	n/a	n/a	*M* = 37.000$ Income (range 20.000–50.000)	*M* = 1 year of college (SD = 2)	n/a
Bovenschen et al. (2016)^9^	46	45.18 (n/a)	n/a	1.2 (0.84)	n/a	n/a	n/a	n/a	n/a
Chesmore et al. (2017)^9^	6,1	n/a	n/a	2,7	both	n/a	n/a	n/a	n/a
Cooley et al. (2015)	n/a	40 (3.98)	n/a	n/a	n/a	n/a			
De Robertis and Litrownik (2004)	n/a	n/a	n/a	n/a	Both	n/a	Median range: 30.000–34.999$	n/a	n/a
De Schipper et al. (2012)^9^	35.12	43.5 (7.1)	n/a	n/a	No	n/a	n/a	n/a	n/a
DeLisle (2011)^9^	n/a	n/a	n/a	n/a	Both	n/a	n/a	n/a	n/a
Denuwelaere and Bracke (2007)^9^	85.2	Foster fathers: 47.6 (4.93) Foster Mothers: 45.7 (5.07)	At least 1	n/a	n/a	n/a	n/a	n/a	n/a
Dubois-Comtois et al. (2015)^9^	20.37	40.7 (8.04)	n/a	n/a	Both	n/a	< 40,000 = 19%, 40–75,000 = 39%, > 75,000 = 42%	72% of foster mothers had a postsecondary education	n/a
Estep (2008)	n/a	45.14 (10.57)	n/a	n/a	Both	Yes	n/a	n/a	n/a
Fuentes et al. (2015)^9^	44.76	47.18 (6.6)	n/a	n/a	n/a	n/a	n/a	33.12% Higher Education Degree, 29.94% Secondary Education, 31.85% Primary Education, 5,1% no formal schooling	n/a
Gabler et al. (2014)^9^	2.73	41 (5.6)	n/a	n/a	n/a	n/a	80% > 2,500.00€	80% at least intermediate school leaving certificate	n/a
Harden et al. (2015)^9^	19	47 (11)	n/a	2 (1.7)	Both	n/a	n/a	College = 62%, High School = 28%, Less than High School = 10%	n/a
Harden et al. (2014)^9^	19	47 (11.6)	n/a	2 (1.9)	Both	n/a	n/a	8% less than high schoo, 29% high school diploma, 63% college	n/a
Harden et al. (2017)^9^	18.8	n/a	n/a	1.95 (1.98)	Both	n/a	> 100.000 = 9%, 60.000–90.000 = 28%, 30.000–59.000 = 17%	Below high school = 10%, high school graduate = 28%, some college or associate's degree = 40%, college graduate = 10%, advanced degree = 10%	n/a
Harpin et al. (2013)^9^	n/a	n/a	n/a	n/a	n/a	n/a	n/a	n/a	n/a
Heywood (2009)^9^	n/a	n/a	n/a	n/a	n/a	n/a	n/a	n/a	n/a
Jacobsen et al. (2018)	15,1	37,7 (5,3)	n/a	1,82	n/a	No	*m* = $100,960	*m* = "high"	n/a
Jones (2004)^9^	n/a	45.6 (n/a)	1.75	n/a	Both	n/a	< 20,000 23%, 21–30 32.7%, 31–4027,4%, 41–50 11%, 51–60 3.5%, 61–70 0.9%	Elementary 1.8%, some high school 8.8%, high school diploma 35.4%, some college 26.5%, college degree 23.9%, graduate school 3.5%	n/a
Kelly (2015)^9^	36	47.56 (8.56)	n/a	3.05 (n/a)	Both	n/a	n/a	n/a	n/a
Leon et al. (2008)^9^	n/a	n/a	n/a	7 (4)	n/a	n/a	n/a	4% less than high school, 23% some high school, 23% high school diploma, 30% college courses, 10% associate's degree, 9% Bachelor, 1% Master	43% employed full-time, 15% employed part-time, 41% unemployed
Linares et al. (2006)^9^	8.6	n/a	n/a	n/a	n/a	n/a	n/a	10.8 (SD = 3.4) completed years of school	n/a
Lindhiem and Dozier (2007)	12.4	46.9 (11.7)	n/a	n/a	n/a	n/a	*m* = $38,000	Mean level of education = 12.6 year	n/a
Migliorini et al. (2016)^9^	n/a	n/a	n/a	n/a	n/a	n/a	n/a	54.54% Senior Secondary School Diplomas, 27.27% University Degrees	n/a
Miller et al. (2019)	72.15	47.21 (8,83)	n/a	n/a	n/a	n/a	n/a	Mean level of Education = 14.89 year	n/a
Olson et al. (2019)^9^	1	n/a	n/a	n/a	n/a	n/a	Median = $45,000	Median = Community College	n/a
Oosterman and Schuengel (2008)^9^	35.4	n/a	n/a	2.23 (1.16)	No	n/a	n/a	n/a	n/a
Perkins (2008)^9^	53.16	n/a	3.26	n/a	Both	n/a	n/a	n/a	n/a
Perkins and Flynn (2008) ^9^	47.4	n/a	3.2	n/a	Both	n/a	n/a	n/a	n/a
Ponciano (2010)^9^	12	n/a	n/a	n/a	n/a	n/a	n/a	n/a	Full-time Employment 18%, Work at home 12%, part-time 16%, not employed 54% (Foster Mothers)
Ponciano (2012)^9^	12	n/a	n/a	1.8 (n/a)	n/a	n/a	n/a	n/a	n/a
Richardson and Gleeson (2012)	n/a	49.66 (n/a)	3.98	n/a	Yes	n/a	< $20,000 = 29%, − 39,999 = 44%, − 59,999 = 18%, 60,000 + = 9%	Less than elementary = 8%, Grade 8 = 24%, High school = 44%, Trade School = 1%, Associate's Degree = 7%, 4 year College = 13%, Graduate = 4%	n/a
Rogers (2016)	Non-kin = 25.97 Kin = 83.21	Non-kin = 56.06 (13.42) Kin = 61.03 (9.68)	Non-kin = 1.45 Kin = 2.68	n/a	Both	n/a	*M* = 36,366 (23,917)	< High School 3%, High School 18.2%, some college 12.1%, Bachelor 54.5%, Advanced 12.1%	Full-time 27.3%, Part-time 3%, Retired 54.5%, Unemployed 15.2%
Salas et al. (2015)^9^	n/a	47.3 (6.6)	n/a	n/a	No	n/a	n/a	n/a	n/a
Sandow (1998)^9^	42	n/a	n/a	1.38 (n/a)	No	Yes	n/a	n/a	n/a
Schofield (2010)	29.9	n/a	n/a	n/a	n/a	n/a	n/a	n/a	n/a
Smith (1994)^9^	22.3	45.2 (n/a)	4.7	1.8 (n/a)	n/a	n/a	Median family income = 35.000	At least high school	n/a
Somers (2010)	n/a	45.09 (n/a)	n/a	n/a	Both	n/a	10,000–24,999 = 4.6%, 25,000–39,999 = 1.9%, $40,000–54,999 = 28.7%, $55,000–69,999 = 2.8%, above $70,000 = 17.6%, Missing = 44,4%	n/a	n/a
Stovall (2001)	n/a	50.55 (11.31)	n/a	n/a	n/a	n/a	< 10,000$ = 15.8%, 10–30,000 = 44.7%, 30–60,000 = 31.6%, > 60,000 = 7.9%	Mean level of education = 7.6 year (SD = 2.1)	n/a
Tucker (2011)^9^	57.6	51.08 (n/a)	n/a	n/a	Both	n/a	n/a	n/a	46.51% currently employed
Vanderfaeillie et al. (2012)^9^	58	48.9 (9.2)	n/a	n/a	Both	n/a	n/a	Higher education 22.4%, higher secondary 40.8%, lower secondary 24.5%, primary 8.1%, missing 4.1%	n/a
Vasileva and Petermann, 2017)^9^	36.98	44.65 (7.34)	n/a	2.33 (1.54)	Both	n/a	n/a	Low education = 9.1%, medium education = 58.7%, high education = 31.8%	n/a
Vuchinich et al. (2002)^9^	n/a	n/a	n/a	n/a	n/a	n/a	n/a	n/a	n/a

^1^Included in meta-analytical process

^2^Times between measurements

^3^Study quality

^4^Examination of additional variables

^5^Sample size of CFC

^6^Included in meta-analytical process

^7^Mean age of CFC in years (SD)

^8^Gender of CFC (percentage male)

^9^Included in meta-analytical process

^10^Length of residence in the actual foster family

^11^Mean age of foster parents in years (SD)

^12^Number of siblings in the foster family

^13^Number of placements of CFC

^14^Professionalism of foster parents

^15^Foster family income

^16^Regarding foster parents

### Parenting Behavior and Children’s Development

For dysfunctional and functional parenting behaviors, separate meta-analyses were computed for variables of child developmental outcomes (Tables 2 and 3).

**Table 2:** Results of the meta-analyses for functional parenting behavior and child development variables

Child development (studies; outcomes)	$\widehat{\theta }$	SE	*t*-Test	*p*		${\rm T}^{2}$	${I}^{2}$	95% CI
Adaptive functioning (6; 40)	0.16	0.05	2.99	0.031	**	0.02	65.85	0.02; 0.30
Externalizing problems (14; 49)	− 0.18	0.07	2.51	0.026	**	0.09	90.64	− 0.33; − 0.02
Internalizing problems (17; 42)	− 0.18	0.05	3.42	0.004	***	0.03	89.32	− 0.29; − 0.07
Attachment security (3; 19)^1^	0.31	0.15	2.10	0.171		0.06	82.96	− 0.32; 0.94
Total problem behavior (4;13)^1^	0.06	0.15	0.42	0.704		0.13	90.69	− 0.41; 0.53

^1^df < 4

*p < 0.10, **p < 0.05, ***p < 0.01

**Table 3:** Results of the meta-analyses for dysfunctional parenting behavior and child development variables

Child development (studies; outcomes)	$\widehat{\theta }$	SE	*t*-Test	*p*		${\rm T}^{2}$	${I}^{2}$	95% CI
Adaptive functioning (5; 11)	− 0.12	0.05	2.36	0.089	*	0.01	46.10	− 0.26; 0.03
Externalizing problems (8; 18)	0.27	0.11	2.54	0.039	**	0.08	91.95	0.19; 0.52
Internalizing problems (8; 16)	0.12	0.05	2.54	0.044	**	0.02	66.11	0.004; 0.24
Attachment security (2; 3)^1^	− 0.07	0.10	0.72	0.603		0.00	0.00	− 1.29; 1.15
Total problem behavior (3; 9)^1^	0.27	0.10	2.89	0.102		0.04	79.40	− 0.13; 0.68

^1^df < 4

*p < 0.10, **p < 0.05, ***p < 0.01

The studies were published between 1994 and 2019. Fifteen studies came from the field of psychology, and twenty-five studies were cross-sectional. The study quality for all 31 studies ranged from 15 to 21.5, and the sample sizes ranged from *n* = 20 to *n* = 5516. As expected, more functional parenting was significantly associated with more adaptive developmental outcomes for CFC (*t* = 2.99, *p* = 0.031), along with less externalizing (*t* = 2.51, *p* = 0.026) and internalizing problems (*t* = 3.42, *p* = 0.004), respectively. Additionally, more dysfunctional parenting behavior was significantly associated with more externalizing (*t* = 2.54, *p* = 0.039) and internalizing problems (*t* = 2.54, *p* = 0.044) for CFC. Note that some random-effects models have less than four degrees of freedom, which indicates few available effect sizes. Therefore, the analysis has low statistical power, and those results must be considered with caution.

The forest plots were inspected and showed similar and expected distributions for all combinations of variables. Forest plots of the associations of parenting behavior and child development variables can be retrieved as supplemental electronic material (Figures E1 to E10).

For reliable moderator analyses, child developmental outcome variables were combined to increase statistical power. Therefore, *adaptive functioning* and *attachment security* were combined to indicate *adaptive child development*, and *internalizing problems*, *externalizing problems*, and *total problem behavior* were combined to indicate *maladaptive child development* (Table 4)*.*

**Table 4:** Results of the meta-analyses for parenting behaviors and summarized child development variables

Functional parenting behavior (studies; variables)	$\widehat{\theta }$	SE	*t*-Test	*p*		${\rm T}^{2}$	${I}^{2}$	95% CI
Adaptive child development (8; 59)	0.18	0.06	3.19	0.016	**	0.03	71.04	0.05; 0.31
Maladaptive child development (19; 104)	− 0.16	0.05	3.17	0.001	***	0.04	89.11	− 0.27; − 0.05
Dysfunctional parenting behavior (studies; variables)								
Adaptive child development (6; 14)	− 0.12	0.04	2.89	0.043	**	0.01	35.06	− 0.23; − 0.01
Maladaptive child development (10; 43)	0.19	0.08	2.33	0.045	**	0.07	89.31	0.01; 0.38

*p < 0.10, **p < 0.05, ***p < 0.01

#### Moderator Analyses for Variables of Functional Parenting Behavior

For functional parenting behavior, sample sizes ranged between 20 and 5516. The studies were published between 1994 and 2017, and the study quality ranged from 15 to 21.5. As shown in Table E3 in the supplemental materials, the association between functional parenting behavior and adaptive child development was significantly higher for longitudinal studies (*t* = 2.41, *p* = 0.049). The rest of the moderator analyses revealed no significant effects. Twenty-one studies with 101 effect sizes were available for the moderator analyses of the association between functional parenting behavior and maladaptive child development. No significant moderator effects could be found.

#### Moderator Analyses for Variables of Dysfunctional Parenting Behavior

Sample sizes ranged between 24 and 439. Studies were published between 1997 and 2019, and the study quality ranged from 15 to 21.5. Table E4 (electronic supplemental material) shows the results of the moderator analyses for adaptive child development. The association between dysfunctional parenting behavior and adaptive child development was significantly less negative when the sample consisted of kinship and nonkinship foster families (*t* = 5.64, *p* = 0.037). Furthermore, the association was less negative when the foster parents’ professionalism had not been reported (*t* = 10.4, *p* = 0.003), or when foster parents had been reported as nonprofessional (*t* > 10, *p* < 0.001). By contrast, the association became more negative (i.e., stronger) when more siblings lived with the actual foster family (*t* > 10, *p* < 0.001). Regarding the moderator analyses for dysfunctional parenting and maladaptive child development, nine studies with 14 effect sizes were available. No significant coefficients were found for the variables.

Please note that some variables could not be analyzed for all effect sizes regarding the moderator analyses due to the number of missing variables. A quantitative summary, therefore, was not possible in these cases.

### Publication Bias

Funnel plot and trim-and-fill methods were conducted with the package *metafor* (Figures E11 to E20, electronic supplementals). This was not possible with the clustered meta-analytical model from *robumeta*, but effect sizes for both methods overall showed the same directions. Therefore, this might be the best estimation. No indices for publication bias were found except for one effect size. For the association between functional parenting behavior and internalizing problem behavior, ten studies were computed as missing on the *left* side (*Z* = − 6.89, *p* < 0.001). However, for this effect size, missing studies on the *right* side would have been expected for publication bias to indicate a trend to zero. Adding the hypothetical effects to the overall effect size would lead to higher associations and would support the reported results. Overall, no evidence was found to support the assumption of unpublished effects with zero or small effect sizes.

### Parenting Style and Children’s Development

The authoritative parenting style could be identified when reviewing the literature for functional parenting styles. Three studies with 13 effect sizes could be interpreted from the literature review. Two studies were from the field of psychology. All studies were cross-sectional and were published between 1999 and 2014. The study quality ranged from 16 to 18, and the sample sizes ranged from 38 to 104. The age range of foster parents was low (45.14–47.18 years) compared to a high age range for the CFC (4.44–11 years^1^). Additionally, the range for the CFC’s length of residence was rather high (22.3–44.76 months). Eight associations between the authoritative parenting style and internalizing, externalizing, and total problem behavior were negative but small (*r* = − 0.13 to *r* = − 0.21), according to Cohen (1988). Four associations were smaller than 0.10 (*r* = − 0.03 to *r* = − 0.07). Associations between the authoritative parenting style and the adaptive functioning of CFC were positive; two were small (*r* = 0.12 and *r* = 0.25), and one only *r* = 0.01. This may indicate that the effect sizes were found to aggregate into a small overall effect (at most).

Authoritarian and permissive parenting styles were identified as dysfunctional parenting styles. Three studies and 21 effect sizes could be used in the literature summary. All studies had a cross-sectional design and were from the field of psychology. They were published between 2008 and 2015, with sample sizes ranging from 33 to 104, and the study quality ranging from 16 to 18. A low range in the ages of CFC (11–13.21 years) could be identified, in comparison to a high range in the length of residence (25.97–83.21 months) and the age of foster parents (45.14–61.03). The authoritarian parenting style was associated slightly positively with internalizing, externalizing, and total problem behavior in CFC in one case (*r* = 0.17) and moderately associated in seven cases (*r* = 0.25 to *r* = 0.42), indicating an overall small to a moderate positive association between the authoritarian parenting style and maladaptive child development outcomes. However, a similar relation was also shown for the authoritarian parenting style and placement stability (*r* = 0.16 and *r* = 0.20 in one study). A permissive parenting style was associated slightly negatively with placement stability in two studies (*r* = − 0.12 and *r* = − 0.18) and, in one case, near zero with *r* = − 0.06. The association with total problem behavior was also very low in one calculation (*r* = − 0.04) and small in three cases (*r* = − 0.15 to *r* = − 0.13). Additionally, the permissive parenting style showed slight positive associations with internalizing problems in three calculations with effect sizes between *r* = 0.13 and *r* = 0.16, and in one effect size close to zero with *r* = − 0.01.

### Parenting Goals and Children’s Development

It was possible to compute effect sizes for functional parenting goals and CFC externalizing and internalizing problem behaviors, respectively, across four studies (Table E5 in the supplemental material). As a result of this, foster parent attachment goals were the most investigated parenting goal variables. However, all associations were not significant.

Three studies examined dysfunctional parenting goals. Specifically, these goals were criticism, rejection, overprotection, and the challenging aspects of being a foster parent (such as looking after a child with special needs). One study showed a medium association with total problem behavior in CFC (*r* = 0.35 and *r* = 0.46) and one study showed a high association between overprotection and placement stability (*r* = 0.99). The challenging aspects of fostering were not associated with externalizing problems for CFC (*r* = − 0.01).

## Discussion

The present study had two primary aims: (1) to identify the associations between foster parents’ parenting and children’s behavior, including the emotional and behavioral development of CFC, and (2) to identify moderators that increase or decrease the strength of these associations. This may help to tailor support systems for foster care.

### Parenting Behavior

Regarding foster parenting behavior, more functional parenting was associated with more adaptive developmental outcomes for CFC and less maladaptive developmental outcomes, respectively. Additionally, more dysfunctional parenting behavior was associated with more maladaptive developmental outcomes for CFC. This shows the potential association of foster parenting behaviors and CFC’s development, especially for functional parenting behavior. It may indicate a buffering or correcting effect of functional parenting behavior for CFC despite former maltreatment or experiences of neglect before foster placement. However, according to the categorization provided by Cohen (1988), all associations were small to moderate, suggesting that many additional variables must be considered when exploring the development of CFC.

When computing possible moderators, it was evident that many variables could not be computed due to a lack of variance in these measures. However, the meta-regression indicated a stronger positive association of functional parenting behavior and adaptive child development in longitudinal studies. This may indicate a stronger relationship between foster parents’ and children’s outcomes over time, resulting in a greater influence on parental behavior. Additionally, CFC may first need to learn adequate reactions and cognitions and adapt to their foster parents’ different parenting behaviors. Foster parents might also need to learn to better adapt their parenting according to the CFC’s special needs.

Looking at the association between dysfunctional parenting behavior and adaptive child development, we found that the minor negative association was weaker for studies with nonprofessional foster parents, or when professionalism was not reported. One reason for this finding might be that parental and familial characteristics (e.g., the age of foster parents, the number of siblings in the family) may have been higher in nonprofessional foster families, or that children with more extreme needs had been selected for placement in foster families with professionals. Therefore, the association between parenting and child development may be moderated more differentially and therefore ended up smaller. The same assumption may apply to kinship states in foster families, where the association was strongest for nonkinship foster families. Furthermore, the negative association between dysfunctional parenting behavior and adaptive child development became stronger when more children lived in the family. This may be because other buffering elements in the relationship between foster parents and children might be reduced, and the dysfunctional characteristics of parenting behavior may more easily interfuse within the parent–child relationship.

In summary, parenting behavior was the best-examined parenting variable in foster family studies, and our meta-analysis supports the association and complex interactions with children’s developmental outcomes. The results could indicate a model of one dimension describing functional and dysfunctional parenting behavior, as Wolfe and McIsaac (2011) described for community samples, because the two linear models could also show one linear effect. However, different potential moderators for the associations were found for functional and dysfunctional parenting behavior. Dysfunctional parenting behavior was also shown to be influenced by more moderator variables. In summary, the results show the high importance of well-elaborated definitions and concrete assessments of parenting behaviors in primary studies. An investigation of the emergence of functional and dysfunctional parenting behaviors here seems to be imperative in further research to address this issue. Additionally, further investigations of parenting behavior dimensions (e.g., responsiveness, demandingness/control, and psychological control; Wolfe and McIsaac 2011) are needed to better distinguish their impact on children’s development. This may also be important for foster parent interventions. Kemmis-Riggs et al. (2018) provided support for foster parent training that promotes functional parenting behaviors.

Many moderators could not be analyzed, even though there is some evidence in former research that other variables influence the association between foster parenting behaviors and the development of CFC. Considering the results of the meta-analysis at hand, the following moderators should be considered in future studies: the professionalism of foster care providers, the kinship status between foster parents and the CFC, the number of children living in the foster family, and the number of placement disruptions of the CFC. Furthermore, more longitudinal studies are necessary to investigate CFC’s development and infer the causes and effects of identified associations.

### Parenting Style

With respect to parenting styles, the results of the current review were mixed. Authoritative and authoritarian parenting styles were mostly related to child development outcomes, the same as one would expect for functional and dysfunctional parenting styles in biological families. These associations were small to moderate, just as they are for parenting behavior. This also applied to permissive parenting style and placement stability, as well as to internalizing problems. Surprisingly, the authoritarian parenting style was also associated with higher placement stability. One could argue that the longer that the children lived in their foster family, this parenting style in foster parents might have been expressed more due to their behavior. Therefore, foster parents may think a more authoritarian style is necessary. On the other hand, authoritarian foster parents may question the continuation of fostering less than parents who exhibit other parenting styles. Furthermore, permissive parenting was—unexpectedly—associated with less externalizing and total problem behavior. An explanation may be that permissive parents do not limit externalizing and total problem behaviors as much as parents with different parenting styles. Therefore, they may *report* those child behaviors less. By contrast, it also may be possible that the CFC truly do show less problematic behavior when experiencing those parenting styles. This is in line with previous research regarding the impact of former maltreatment and experiences of neglect (e.g., Bruce et al. 2009; Price and Landsverk 1998). By this, a permissive or authoritarian parenting style would be better tailored to the expectations, and therefore reactions, of the CFC (e.g., da Silva Ferreira et al. 2014). However, the limited evidence of associations between parenting styles and the developmental paths of CFC makes it difficult to explain those rather counter-intuitive findings. Goemans et al. (2015) found little impact on the course of behavioral or emotional problems in CFC, which may be explained by the long-term altering effects of maltreatment experiences on functional and structural brain development (e.g., Teicher et al. 2002). This is still a mostly uninvestigated area in previous research, and further investigation is highly needed.

### Parenting Goals

Regarding parenting goals, we found no significant effects between functional parenting goals and internalizing and externalizing problems with CFC. Only three studies investigated dysfunctional parenting goals. Additionally, one of the effect sizes the research discovered was very high (*r* = 0.99). Therefore, this seems to be an area that is less investigated than that of parenting behaviors and styles. Thus, the representativity for parenting goals and child development may be questionable. Additionally, parenting goals may only have an indirect influence on child developmental variables. Thus, it is more difficult for researchers to define associations, and the probability of publication may be reduced. However, this should not be an excuse for neglecting the potential impact of this dimension on the development of CFC and should be investigated in future research.

### The Comparison to Associations in Biological Families

Regarding the associations between foster parenting behavior and CFC’s developmental outcomes, our findings were generally in line with those reported previously for biological families (Table E6 in the supplemental material). Therefore, one could assume that the associations of parenting and children’s developmental outcomes are similar in foster and biological families. This may be a promising result because similar parenting interventions might also help foster parents improve the developmental outcomes of the children in their care. However, a descriptive comparison of the effect sizes may lead to the false assumption that the underlying processes are comparable in both types of families. For example, each family type’s different composition may lead to different moderating effects, as noted above. As a result, the effect sizes may be similar but also moderated by unrelated variables. Additionally, there is a high variance in effect sizes for these results. This may support the assumption that the relationship between foster parenting behavior and the development of CFC runs in the same direction as it does in biological families but is also influenced by additional or different moderators (for example, the number of placements of the CFC beforehand). Therefore, further research is needed to better distinguish between parenting and child development associations in biological and foster families. This goes along with Kemmis-Riggs et al. (2018), who identified that specific intervention components focusing on the special needs of CFC were effective. However, the differential susceptibility hypothesis (e.g., Pluess and Belsky 2010) may argue that CFC, who are most susceptible to environmental adversity, may also benefit most from developmentally supportive rearing conditions. Unfortunately, for the meta-analysis at hand, it was not possible to distinguish between the groups of potentially more or less susceptible children. Hence, this is another area that should be investigated in future research.

Additionally, the parenting style results, as combinations of parenting behavioral dimensions, show similar effects only for authoritative parenting styles. This may indicate that this parenting style is favored in the training of foster parents. The current evidence is rather limited concerning drawing firm conclusions, however. For instance, the influence of the foster parenting style and goals, especially, could not be meta-analytically computed entirely due to the lack of data. Future research should better differentiate those concepts to understand possible associations. In addition, the bidirectional character of the association, as stated for biological families (Brenner and Fox 1999), must be examined in more detail.

### Limitations

Several effects could not be computed for the meta-analysis at hand due to a lack of study variance or reports in primary studies. This is particularly related to foster parenting goals and styles. We tried to counter this by describing the few effects that could be found. For some analyses, models showed a degree of freedom smaller than four. Those results may be biased, and therefore, should be interpreted with caution. Merging the variables of CFC into adaptive and maladaptive child developmental outcomes may have masked effects of less global and more specific child variables (e.g., the impact on internalizing and externalizing problem behavior may be influenced by different parenting behaviors). However, moderator analyses could only be conducted when these variables were accumulated because, otherwise, there would not have been a large enough effect size. Additionally, regarding the exclusion of primary studies, a large body of research (*k* = 38 studies) had to be excluded due to a lack of quantitative information. On the one hand, this could indicate a rather conservative estimation of effect sizes for the analyses at hand. On the other hand, it shows the high need to report unbiased effect sizes, such as correlation tables.

Furthermore, we chose to compute the interrater agreement instead of more conservative measurements. The coding sheet contained numeric, ordinal, nominal, and open-answer variables. It was, therefore, not possible to select a single statistical measurement for interrater reliability. Additionally, it is unlikely that coding decisions were made by chance because of the high number of open-answer variables. Therefore, a conservative interrater reliability measurement may not be necessary. The two variables with the lowest agreement (86.94% and 75.80%, respectively) were areas of child development and parenting, although the agreement still can be interpreted as satisfactory. In future research, a clearer definition of these central variables would be helpful to increase the comparability of different studies and research approaches.

Moreover, 21 studies could not be retrieved, and the full text of these could not be screened for suitability with the meta-analysis and review. This should be noted in terms of publication bias, even though publication bias testing did not flag this as an issue. Finally, it should be noted that we decided to include the attachment security of CFC in the analysis while searching for eligible studies because of the large appearance of those variables. However, we did not specify these terms in the database searches. Therefore, we cannot guarantee that all studies with eligible research questions for CFC attachment security were found.

## Conclusion

The present literature review and meta-analysis examined the association between foster parents’ parenting and children’s developmental outcomes in foster care. It can be concluded that the main effects of foster parenting behavior and children’s development are in line with previous findings in biological families. However, differences could be found, especially for functional parenting, which may have a higher impact on the development of CFC. This may help plan foster family placements and interventions for foster parents because of the special needs of CFC. Regarding the potential influence that foster parents have on CFC, more studies, especially longitudinal studies, should investigate not only the associations but also the causal effects and possible moderators, as noted above.

## Supplementary Information

Below is the link to the electronic supplementary material.

Electronic supplementary material 1 (DOCX 548 kb)

Electronic supplementary material 2 (DOCX 377 kb)

Electronic supplementary material 3 (DOCX 13 kb)

Electronic supplementary material 4 (DOCX 17 kb)

Electronic supplementary material 5 (DOCX 14 kb)

Electronic supplementary material 6 (DOCX 15 kb)

Electronic supplementary material 7 (DOCX 15 kb)

Electronic supplementary material 8 (DOCX 14 kb)

## Footnotes

1 Decimal places of the numbers are different because of different reports in the primary studies.
