# Supplementary material for: Foster Parents’ Parenting and the Social-Emotional Development and Adaptive Functioning of Children in Foster Care: A PRISMA-Guided Literature Review and Meta-Analysis
Source: Clin Child Fam Psychol Rev. 2021 Feb 16;24(2):326–47. doi: 10.1007/s10567-020-00336-y (PMC8131300; doi:10.1007/s10567-020-00336-y)
Supplement: Supplementary file 1 — Electronic supplementary material 1 (DOCX 548 kb) [file 10567_2020_336_MOESM1_ESM.docx]

**
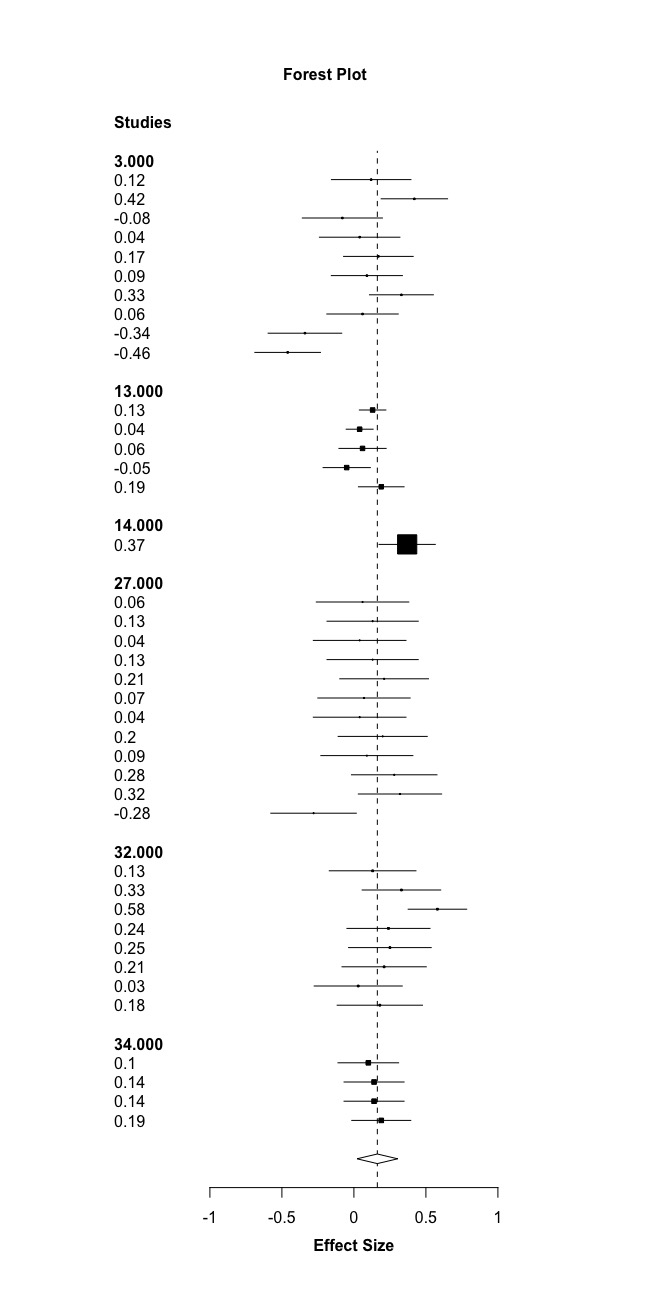
**

**Tucker, 2010**

**Sandow, 1997**

**Smith, 1994**

**Ponciano, 2012**

**Perkins, 2008; Perkins & Flynn, 2009**

**Harden et al., 2014; Harden et al., 2017**

**Association of functional parenting behavior and adaptive functioning**

**Figure E1.** Forest plot of the effect sizes for clustered studies of functional parenting behavior and adaptive functioning.

**
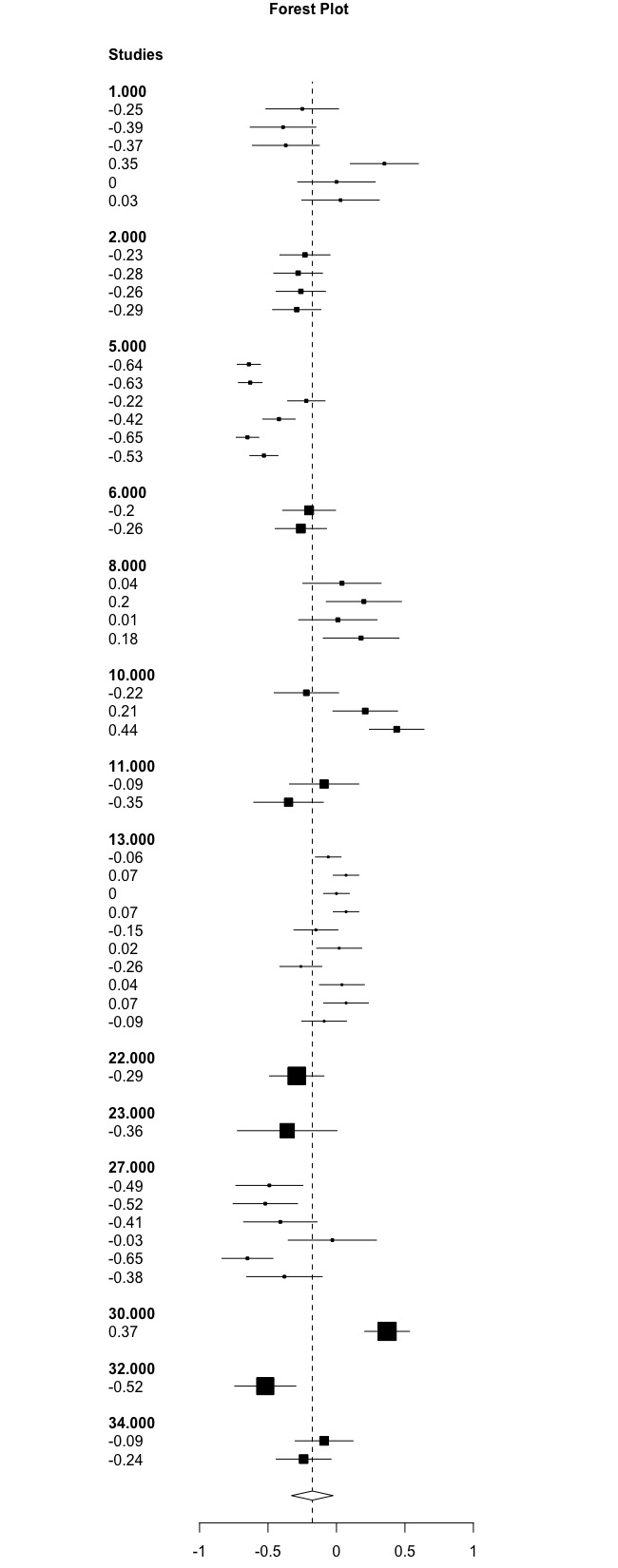
**

**Effect Size**

**Association of functional parenting behavior and externalizing problems**

**Vanderfaeillie et al., 2012**

**DeLisle, 2010**

**Denuwelaere & Bracke, 2007**

**Gabler et al., 2014**

**Fuentes, et al., 2014; Salas et al., 2015**

**Linares et al., 2006**

**Oosterman & Schuengel, 2008**

**Perkins, 2008; Perkins & Flynn, 2009**

**Dubois-Comtois et al., 2015**

**Vuchinich et al., 2002**

**Smith, 1994**

**Jones, 2004**

**Sandow, 1997**

**Tucker, 2010**

**Figure E2**. Forest plot of the effect sizes for clustered studies of functional parenting behavior and externalizing problems.

**
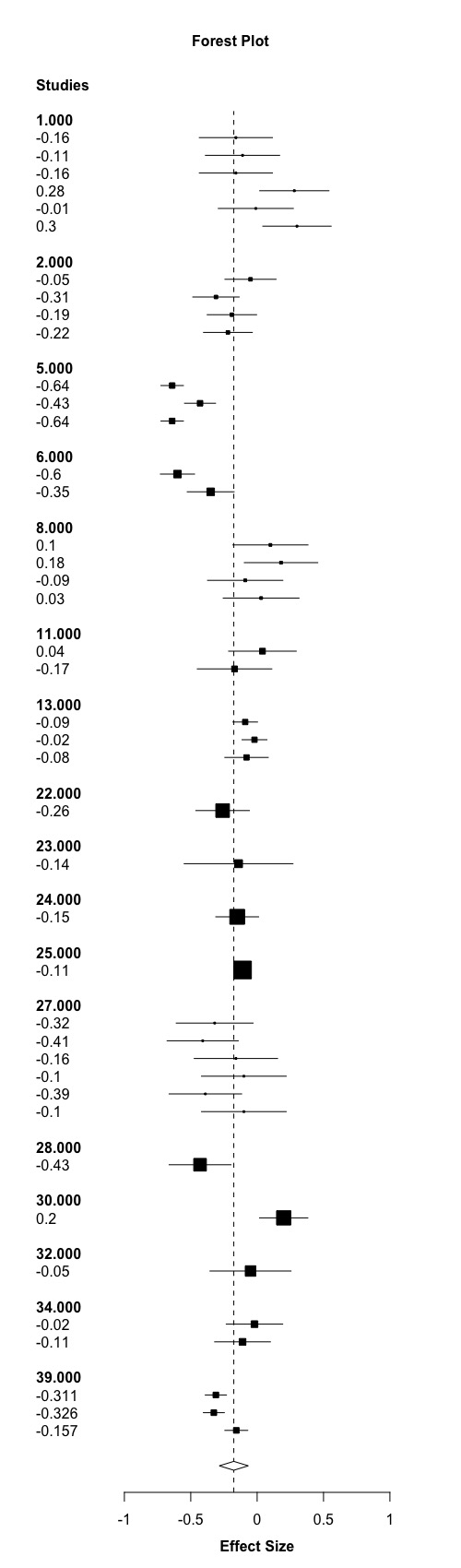
**

**Chesmore et al., 2017**

**Fuentes, et al., 2014**

**Tucker, 2010**

**Sandow, 1997**

**Jones, 2004**

**Migliorini et al., 2015**

**Smith, 1994**

**Harpin et al., 2013**

**Leon et al., 2008**

**Vuchinich et al., 2002**

**Dubois-Comtois et al., 2015**

**Perkins, 2008; Perkins & Flynn, 2009**

**Oosterman & Schuengel, 2008**

**Gabler et al., 2014**

**Denuwelare & Bracke, 2007**

**DeLisle, 2010**

**Vanderfaeillie et al., 2012**

**Association of functional parenting behavior and internalizing problems**

**Figure E3**. Forest plot of the effect sizes for clustered studies of functional parenting behavior and internalizing problems.


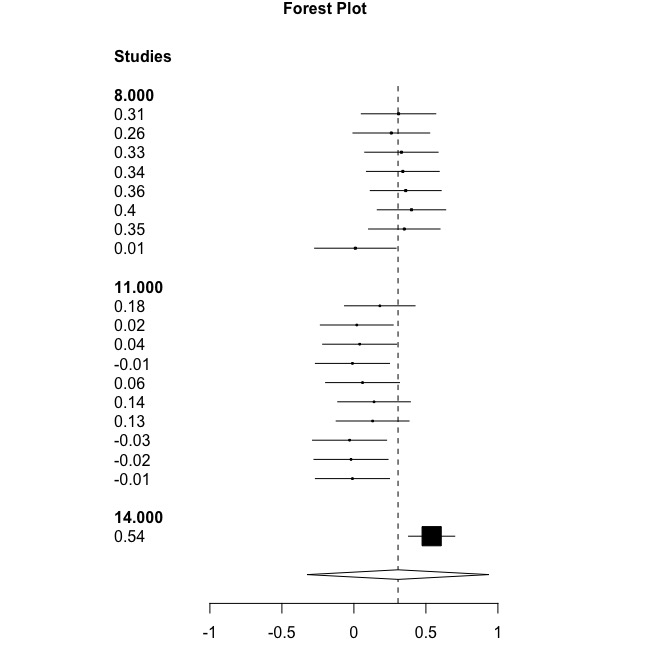


**Effect Size**

**Ponciano, 2010**

**De Schipper et al., 2012; Oosterman & Schuengel, 2008**

**Bovenschen et al., 2016; Gabler et al. 2014**

**Association of functional parenting behavior and attachment security**

**Figure E4**. Forest plot of the effect sizes for clustered studies of functional parenting behavior and attachment security.


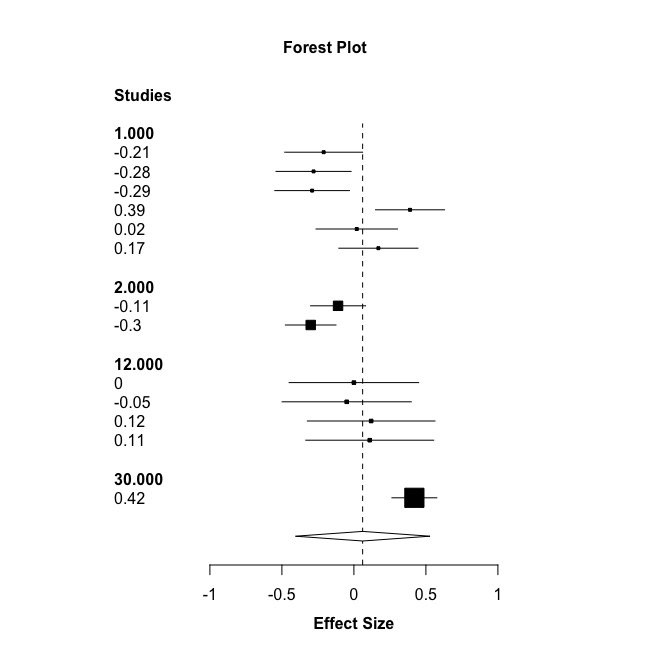


**Association of functional parenting behavior and total problem behavior**

**Jones, 2004**

**Kelly, 2015**

**Salas et al., 2015**

**Vanderfaeillie et al., 2012**

**Figure E5**. Forest plot of the effect sizes for clustered studies of functional parenting behavior and total problem behavior.

**
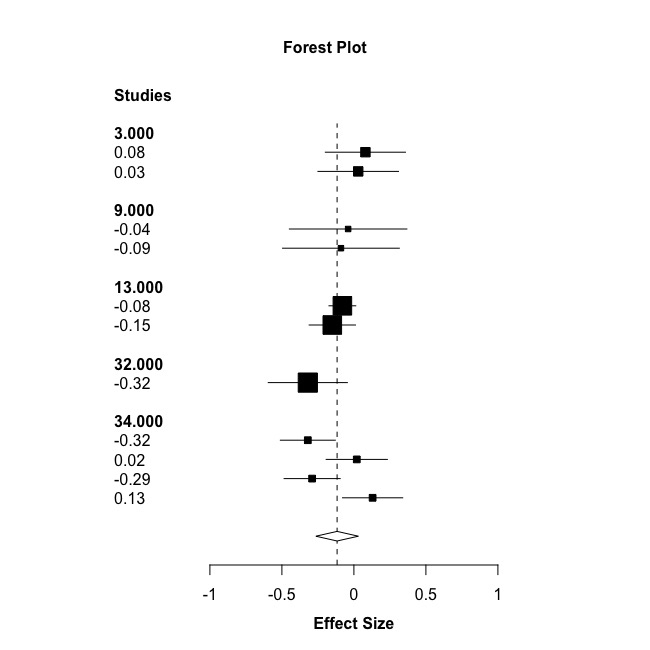
**

**Association of dysfunctional parenting behavior and adaptive functioning**

**Tucker, 2010**

**Sandow, 1997**

**Perkins, 2008; Perkins & Flynn, 2009**

**Heywood, 2009**

**Harden et al., 2017**

**Figure E6**. Forest plot of the effect sizes for clustered studies of dysfunctional parenting behavior and adaptive functioning.


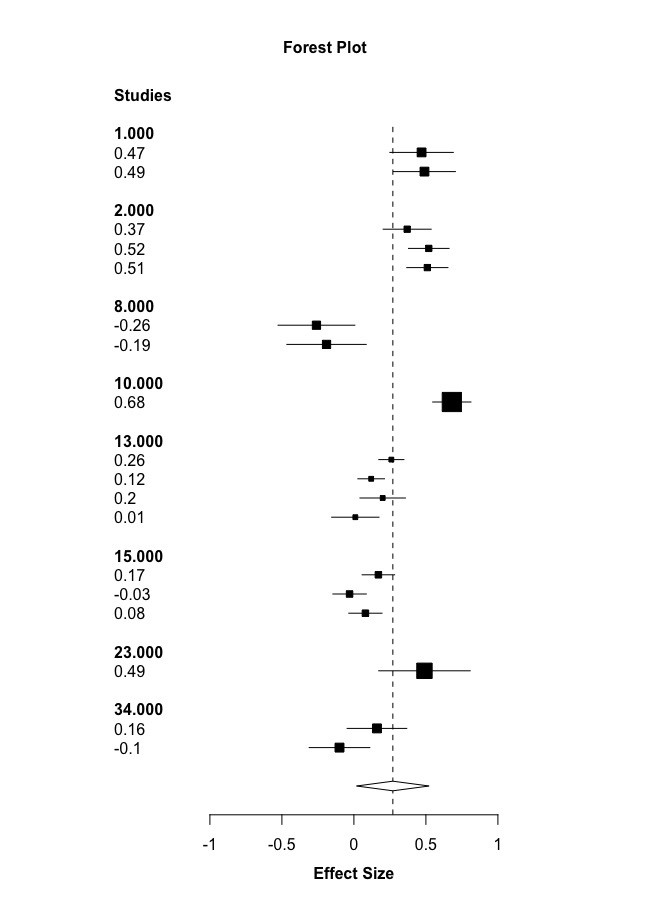


**Perkins, 2008; Perkins & Flynn, 2009**

**Tucker, 2010**

**Vuchinich et al., 2002**

**Vasileva & Petermann, 2017**

**Linares et al., 2006**

**Gabler et al., 2014**

**Fuentes et al., 2014**

**Vanderfaeillie et al., 2012**

**Association of dysfunctional parenting behavior and externalizing problems**

**Figure E7**. Forest plot of the effect sizes for clustered studies of dysfunctional parenting behavior and externalizing problems.


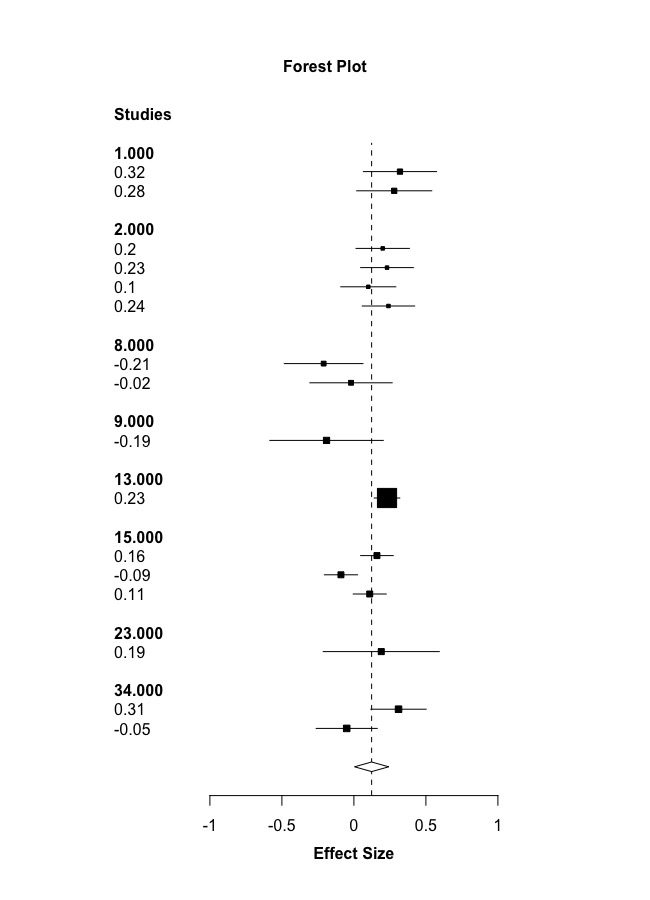


**Association of dysfunctional parenting behavior and internalizing problems**

**Perkins, 2008; Perkins & Flynn, 2009**

**Tucker, 2010**

**Vuchinich et al., 2002**

**Vasileva & Petermann, 2017**

**Heywood, 2009**

**Gabler et al., 2014**

**Fuentes et al., 2014**

**Vanderfaeillie et al., 2012**

**Figure E8**. Forest plot of the effect sizes for clustered studies of dysfunctional parenting behavior and internalizing problems.


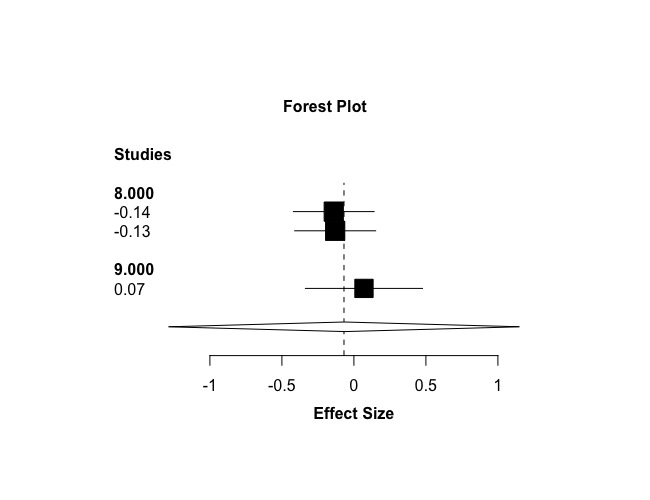


**Heywood, 2009**

**Gabler et al., 2014**

**Association of dysfunctional parenting behavior and attachment security**

**Figure E9**. Forest plot of the effect sizes for clustered studies of dysfunctional parenting behavior and attachment security.

**Association of dysfunctional parenting behavior and total problem behavior**

**
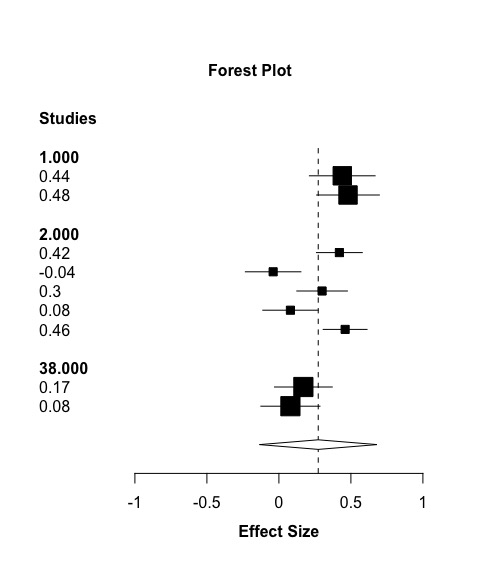
**

**Olson et al., 2019**

**Fuentes et al., 2014; Salas et al., 2015**

**Vanderfaeillie et al., 2012**

**Figure E10**. Forest plot of the effect sizes for clustered studies of dysfunctional parenting behavior and total problem behavior.
