# Supplementary material for: Foster Parents’ Parenting and the Social-Emotional Development and Adaptive Functioning of Children in Foster Care: A PRISMA-Guided Literature Review and Meta-Analysis
Source: Clin Child Fam Psychol Rev. 2021 Feb 16;24(2):326–47. doi: 10.1007/s10567-020-00336-y (PMC8131300; doi:10.1007/s10567-020-00336-y)
Supplement: Supplementary file 2 — Electronic supplementary material 2 (DOCX 377 kb) [file 10567_2020_336_MOESM2_ESM.docx]

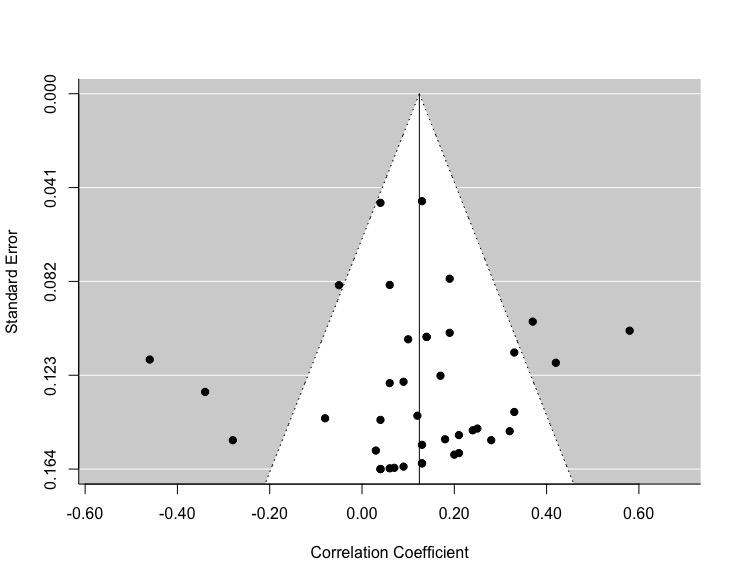
 **Figure E11**. Funnel plot of the effect sizes for functional parenting behavior and adaptive functioning.

**
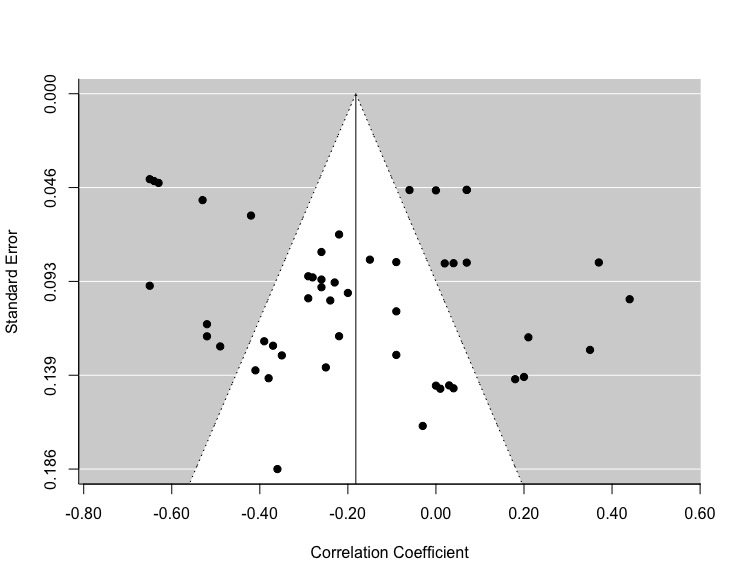
**

**Figure E12**. Funnel plot of the effect sizes for functional parenting behavior and externalizing problems.


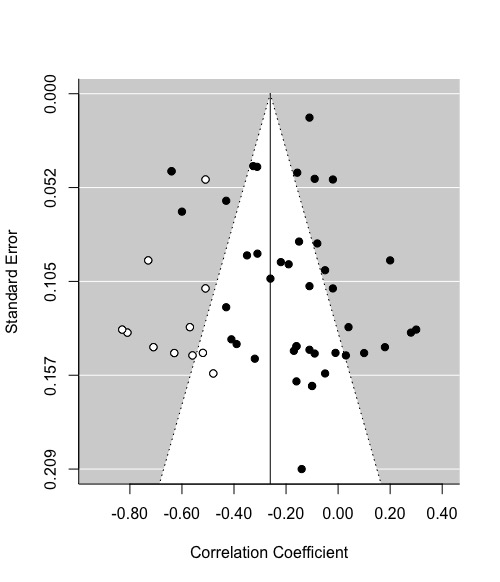


**Figure E13**. Funnel plot of the effect sizes for functional parenting behavior and internalizing problems.


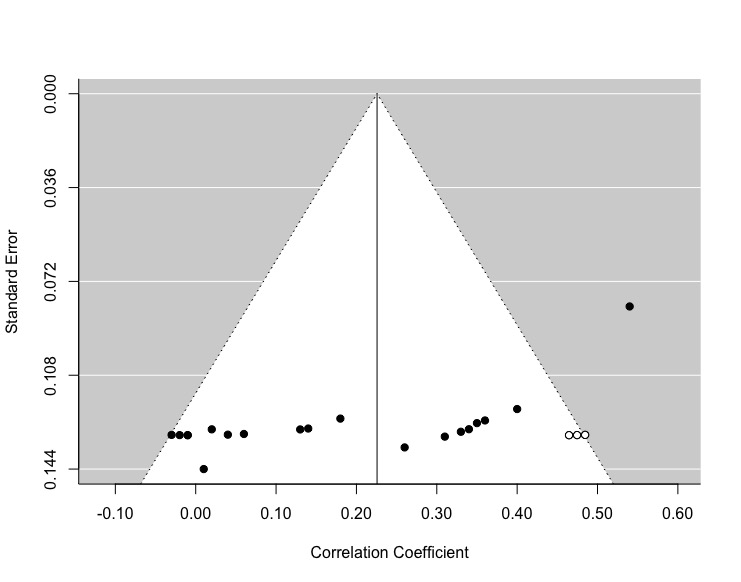


**Figure E14**. Funnel plot of the effect sizes for functional parenting behavior and attachment security.


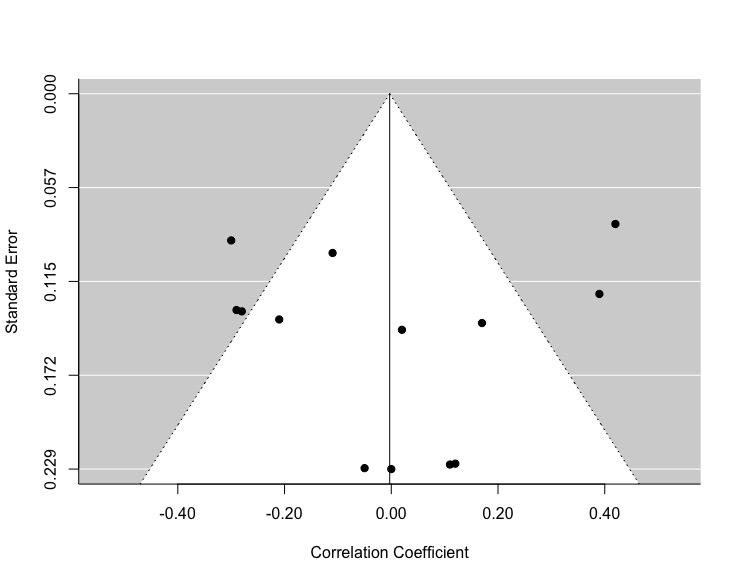


**Figure E15**. Funnel plot of the effect sizes for functional parenting behavior and total problem behavior.


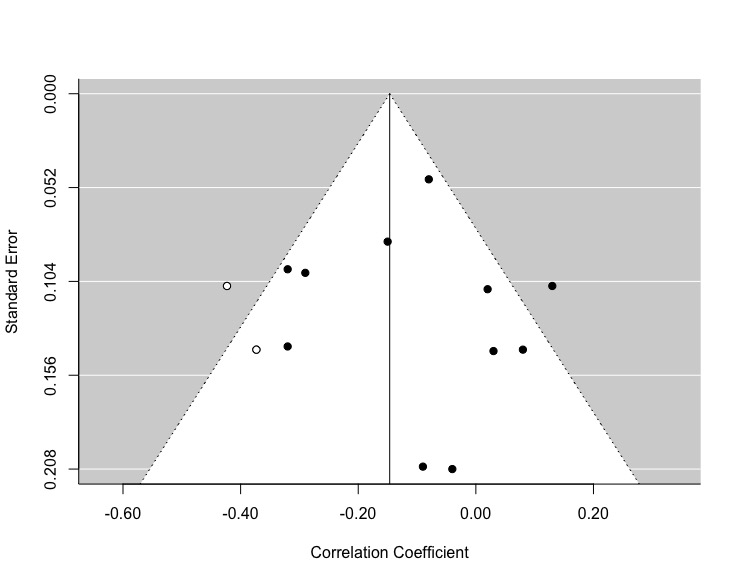


**Figure E16**. Funnel plot of the effect sizes for dysfunctional parenting behavior and adaptive functioning.


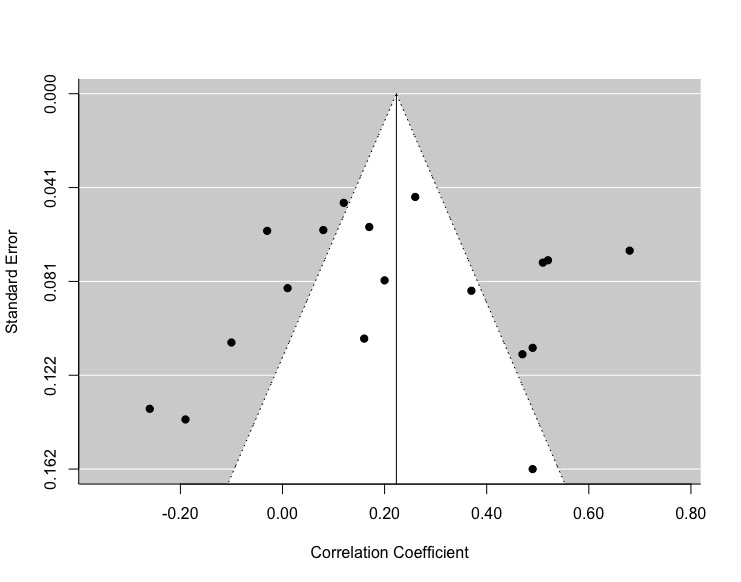


**Figure E17**. Funnel plot of the effect sizes for dysfunctional parenting behavior and externalizing problems.


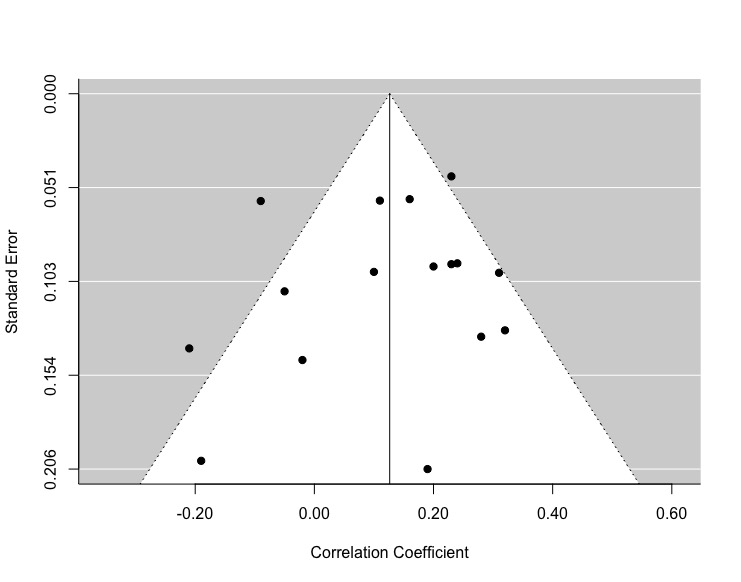


**Figure E18**. Funnel plot of the effect sizes for dysfunctional parenting behavior and internalizing problems.


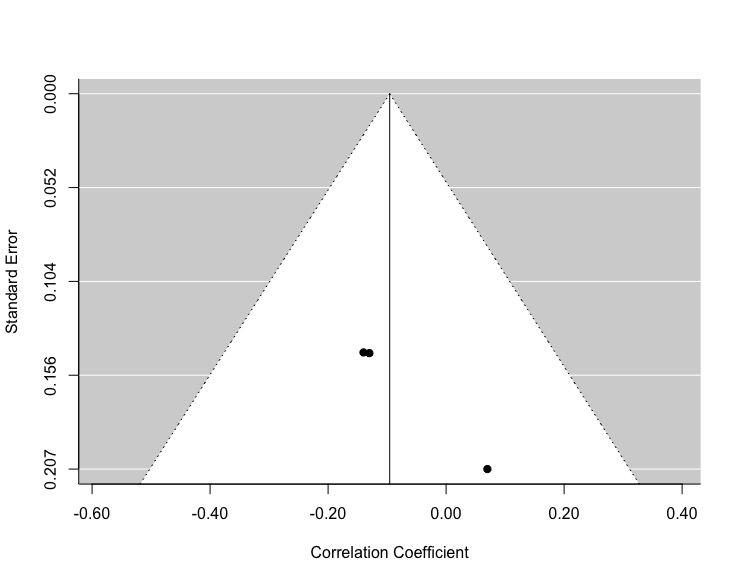


**Figure E19**. Funnel plot of the effect sizes for dysfunctional parenting behavior and attachment security.


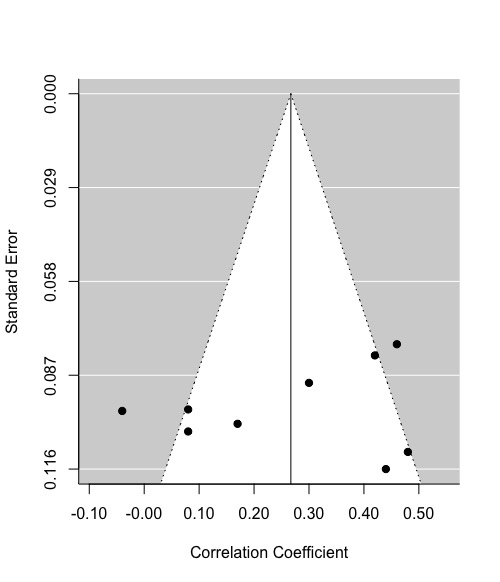


**Figure E20**. Funnel plot of the effect sizes for dysfunctional parenting behavior and total problem behavior.
