# Supplementary material for: Foster Parents’ Parenting and the Social-Emotional Development and Adaptive Functioning of Children in Foster Care: A PRISMA-Guided Literature Review and Meta-Analysis
Source: Clin Child Fam Psychol Rev. 2021 Feb 16;24(2):326–47. doi: 10.1007/s10567-020-00336-y (PMC8131300; doi:10.1007/s10567-020-00336-y)
Supplement: Supplementary file 3 — Electronic supplementary material 3 (DOCX 13 kb) [file 10567_2020_336_MOESM3_ESM.docx]

**Table E1.** Means, standard deviations, and ranges of included variables on study level.

| **Variable** | ***Mean*** | ***SD*** | ***Median*** | **Range** |
| --- | --- | --- | --- | --- |
| Study Quality  (N=43) | 17.30 | 1.95 |  | 12 – 21.5 |
| Sample Size Foster Parents*  (N=43) | 223.92 | 831.80 | 75 | 20 – 5,516 |
| Sample Size CFC *  (N=43) | 222.36 | 832.10 | 64 | 20 – 5,516 |
| Age of CFC in years  (N = 40) | 7.41 | 4.30 |  | 1.00 – 15.00 |
| Gender of CFC (percentage male)  (N = 37) | 51.05 | 7.53 |  | 36.10 – 73.00 |
| length of residence in months  (N = 29) | 31.82 | 21.47 |  | 1.00 – 85.00 |
| Age of foster parents in years  (N = 24) | 46.22 | 4.19 |  | 38.00 – 59.00 |
| Number of children living in foster family  (N = 7) | 3.85 | 1.31 |  | 1.00 – 5.00 |
| Number of placements of CFC  (N = 13)* | 2.40 | 1.47 | 2 | 1.00 – 7.00 |

*Median additionally was computed because of outliers.
