# Supplementary material for: Foster Parents’ Parenting and the Social-Emotional Development and Adaptive Functioning of Children in Foster Care: A PRISMA-Guided Literature Review and Meta-Analysis
Source: Clin Child Fam Psychol Rev. 2021 Feb 16;24(2):326–47. doi: 10.1007/s10567-020-00336-y (PMC8131300; doi:10.1007/s10567-020-00336-y)
Supplement: Supplementary file 4 — Electronic supplementary material 4 (DOCX 17 kb) [file 10567_2020_336_MOESM4_ESM.docx]

**Table E2.** Characteristics and proportions of included variables on study level (*N*=43).

| **Variable** | **Characteristics** | **Proportions** |
| --- | --- | --- |
| Science Area | Psychology  Other | 55.81%  44.19% |
| Country of Study | USA  Other | 67.44%  32.56% |
| Publication Type | Journal Article  Dissertation | 74.42%  25.58% |
| Study design | Cross-sectional  longitudinal | 79.07%  20.93% |
| Type of measurement | Association  B-weight  T-value  Cohen’s d | 69.77%  25.58%  2.33%  2.33% |
| Examination of additional variables | Yes  No | 79.07%  20.93% |
| Area(s) of child development^1^ | Adaptive functioning  Attachment security  Internalizing problems  Externalizing problems  Total problem behavior  Placement stability | 25.58%  18.60%  46.51%  51.16%  20.93%  9.30% |
| Information source(s) of child development^1^ | Behavioral observation  External report  Self report | 13.95%  74.42%  20.93% |

**Table 3.** Characteristics and proportions of included variables on study level (*N*=38). *continued*

| **Variable** | **Characteristics** | **Proportions** |
| --- | --- | --- |
| Area(s) of foster parenting^1^ | Parenting behavior  Parenting style  Parenting goals | 72.09%  9.30%  27.91% |
| Information source(s) of foster parenting^1^ | Behavioral observation  External report  Self report | 27.91%  13.95%  60.47% |
| Kin Foster Care | No  Yes  Both  Not reported | 9.30%  2.33%  39.53%  32.56% |
| Professionalism | Yes  No  Not reported | 4.56%  2.33%  93.02% |
| Foster Family Income | Reported  Not reported | 34.88%  65.12% |
| Highest education of foster parents | Reported  Not reported | 51.16%  48.84% |
| Employment state of foster parents | Reported  Not reported | 11.63%  88.37% |

^1^Multiple selections possible.
