# Supplementary material for: Foster Parents’ Parenting and the Social-Emotional Development and Adaptive Functioning of Children in Foster Care: A PRISMA-Guided Literature Review and Meta-Analysis
Source: Clin Child Fam Psychol Rev. 2021 Feb 16;24(2):326–47. doi: 10.1007/s10567-020-00336-y (PMC8131300; doi:10.1007/s10567-020-00336-y)
Supplement: Supplementary file 5 — Electronic supplementary material 5 (DOCX 14 kb) [file 10567_2020_336_MOESM5_ESM.docx]

**Table E3.** Results of the moderator analyses for functional parenting behaviors and adaptive child development.

|  |  | Regression coefficient | *SE* | *t-Test* | *p* | | *95%-CI* |
| --- | --- | --- | --- | --- | --- | --- | --- |
| Study design | Cross-sectional (Intercept) | 0.17 | 0.06 | 2.95 | 0.023 | ** | 0.03; 0.31 |
|  | Longitudinal | 0.14 | 0.06 | 2.41 | 0.049 | ** | <0.01; 0.28 |
| Number of placements^1^ |  | -0.20 | 0.06 | 3.40 | 0.07 | * | -0.43; 0.04 |

*Notes.* Eight studies and 59 effect sizes were used in the analyses. Intercept is only reported for dummy-coded (nominal) variables. For numerical variables, intercept was always the main association reported above. Only significant effect sizes are reported in the table.

*p < 0.10, **p < 0.05, ***p < 0.01

^1^df < 4
