# Supplementary material for: Foster Parents’ Parenting and the Social-Emotional Development and Adaptive Functioning of Children in Foster Care: A PRISMA-Guided Literature Review and Meta-Analysis
Source: Clin Child Fam Psychol Rev. 2021 Feb 16;24(2):326–47. doi: 10.1007/s10567-020-00336-y (PMC8131300; doi:10.1007/s10567-020-00336-y)
Supplement: Supplementary file 6 — Electronic supplementary material 6 (DOCX 15 kb) [file 10567_2020_336_MOESM6_ESM.docx]

**Table E4.** Results of the moderator analyses for dysfunctional parenting behaviors and adaptive child development.

|  |  | Regression coefficient | *SE* | *t-Test* | *p* | | *95%-CI* |
| --- | --- | --- | --- | --- | --- | --- | --- |
| Year of publication^1^ |  | 0.02 | <0.01 | 4.56 | 0.069 | * | -0.003; 0.04 |
| Professionalism of foster parents^1^ | Yes (Intercept) | -0.32 | <0.01 | >10 | <0.001 | *** | -0.32; -0.32 |
|  | No | 0.35 | <0.01 | >10 | <0.001 | *** | 0.35; 0.35 |
|  | Not reported | 0.23 | 0.02 | 10.4 | 0.003 | *** | 0.15; 0.30 |
| Kin foster care^1^ | No | -0.32 | <0.01 | >10 | <0.001 | *** | -0.32; -0.32 |
|  | Both | 0.24 | 0.04 | 5.64 | 0.037 | ** | 0.04; 0.45 |
|  | Not reported | 0.23 | 0.06 | 4.10 | 0.152 |  | -0.48; 0.93 |
| Number of siblings |  | -1.17 | <0.01 | >10 | <0.001 | *** | -1.17; -1.17 |

*Notes.* Six studies and 14 effect sizes were used in the analyses. Intercept is only reported for dummy-coded (nominal) variables. For numerical variables, intercept was always the main association reported above. Only significant effect sizes are reported in the table.

*p < 0.10, **p < 0.05, ***p < 0.01

^1^df < 4
