# Supplementary material for: Foster Parents’ Parenting and the Social-Emotional Development and Adaptive Functioning of Children in Foster Care: A PRISMA-Guided Literature Review and Meta-Analysis
Source: Clin Child Fam Psychol Rev. 2021 Feb 16;24(2):326–47. doi: 10.1007/s10567-020-00336-y (PMC8131300; doi:10.1007/s10567-020-00336-y)
Supplement: Supplementary file 7 — Electronic supplementary material 7 (DOCX 15 kb) [file 10567_2020_336_MOESM7_ESM.docx]

**Table E5.** Results of the meta-analyses for functional parenting goals and child development variables.

| Child Development  (Studies; Outcomes) | $\hat{\theta}$ | *SE* | *t-Test* | *p* | | $T^{\boldsymbol{2}}$ | $I^{\boldsymbol{2}}$ | *95%-CI* |
| --- | --- | --- | --- | --- | --- | --- | --- | --- |
| Externalizing Problems  (4; 10)^1^ | -0.11 | 0.20 | 0.52 | 0.64 |  | 0.16 | 92.14 | -0.75; 0.54 |
| Internalizing Problems  (3; 14) | -0.19 | 0.07 | 2.74 | 0.113 |  | 0.01 | 28.92 | -0.52; 0.12 |

^1^df < 4.

*p < 0.10, **p < 0.05, ***p < 0.01
