# Supplementary material for: Foster Parents’ Parenting and the Social-Emotional Development and Adaptive Functioning of Children in Foster Care: A PRISMA-Guided Literature Review and Meta-Analysis
Source: Clin Child Fam Psychol Rev. 2021 Feb 16;24(2):326–47. doi: 10.1007/s10567-020-00336-y (PMC8131300; doi:10.1007/s10567-020-00336-y)
Supplement: Supplementary file 8 — Electronic supplementary material 8 (DOCX 14 kb) [file 10567_2020_336_MOESM8_ESM.docx]

**Table E6.** Comparison of effect sizes for the meta-analysis at hand and meta-analyses for biological families.

| Child Development Variables | Functional parenting behavior ($\hat{\theta}$) | Dysfunctional parenting behavior ($\hat{\theta}$) | Effects in biological families |
| --- | --- | --- | --- |
| Adaptive Functioning | 0.16 | -0.12 | .08 (fp) & -.14 (dp)^1^; .04-.17 (fp) & -.11 - -.23 (dp)^2^ |
| Externalizing Problems | -0.18 | 0.27 | .24 (inverted for fp)^3^; -.11 - -.19 (fp) & .08 - .22 (dp)^4^ |
| Internalizing Problems | -0.17 | 0.12 | .21 (dp)^5^; .28 (dp)^6^ |
| Attachment Security | 0.31 | -0.07 | .22 (fp)^7^ |

*Notes.* fp = functional parenting, dp = dysfunctional parenting.

^1^Karreman et al. (2006)

^2^Pinquart (2015)

^3^Rothbaum & Weisz (1994)

^4^Pinquart (2017)

^5^McLeod, Wood, & Weisz (2007)

^6^McLeod, Weisz, & Wood (2007)

^7^De Wolff & van Ijzendoorn (1997)
